# Prognostic and tumor microenvironmental features of gastric cancer revealed by macrophage polarization and protein lactylation-related genes

**DOI:** 10.3389/fgene.2025.1541489

**Published:** 2025-07-02

**Authors:** Zifan Xu, Zi Lei, Shilan Peng, Sha Li, Dehui Kong, Hongqiong Duan, Man Zhang, Guomiao Su, Guoqing Pan

**Affiliations:** ^1^ Department of Pathology, Kunming Medical University, Kunming, Yunnan, China; ^2^ Department of Pathology, First Affliated Hospital of Kunming Medical University, Kunming, China

**Keywords:** gastric cancer, prognostic genes, macrophage polarization, protein lactylation, bioinformatics analysis

## Abstract

**Background:**

The progression of gastric cancer (GC) is closely linked to macrophage polarization and protein lactylation; however, its underlying mechanisms remain poorly understood. This study aimed to elucidate the molecular mechanisms of GC using transcriptomic analysis.

**Methods:**

Candidate genes were identified by intersecting differentially expressed genes with key module genes associated with protein lactylation and macrophage polarization. Protein-protein interaction analysis was performed to uncover interacting genes. Prognostic genes were determined using univariate Cox regression and machine learning techniques, with model accuracy assessed via training and validation datasets. Further, enrichment analysis, immune infiltration profiling, gene mutation analysis, and drug sensitivity assessments were conducted for high- and low-risk groups. Chromosomal localization, gene-gene interaction network analysis, and expression validation of prognostic genes were also performed.

**Results:**

Two prognostic genes, ERCC6L and MYB, were identified as significant markers of prognosis through comprehensive analyses. A risk model based on these genes accurately predicted survival in patients with GC. Enrichment analysis revealed pathways such as the muscle myosin complex and adipogenesis as significantly involved in GC. Immune infiltration analysis identified 13 immune cell types, including monocytes, with strong associations to the prognostic genes. TTN, TP53, and MUC16 exhibited the highest mutation rates in both risk groups. Drug sensitivity analysis highlighted AZD.0530, CCT007093, DMOG, JNJ.26854165, and LFM.A13 as promising therapeutic candidates. ERCC6L is located on chromosome X, while MYB is located on chromosome 6. Gene-gene interaction network analysis revealed interactions between prognostic genes and other key genes. In both datasets, expression of prognostic genes was significantly higher in the GC cohort.

**Conclusion:**

This study identified ERCC6L and MYB as key prognostic genes, facilitating the development of a risk model that offers novel insights into potential therapeutic strategies for GC.

## Graphical Abstract

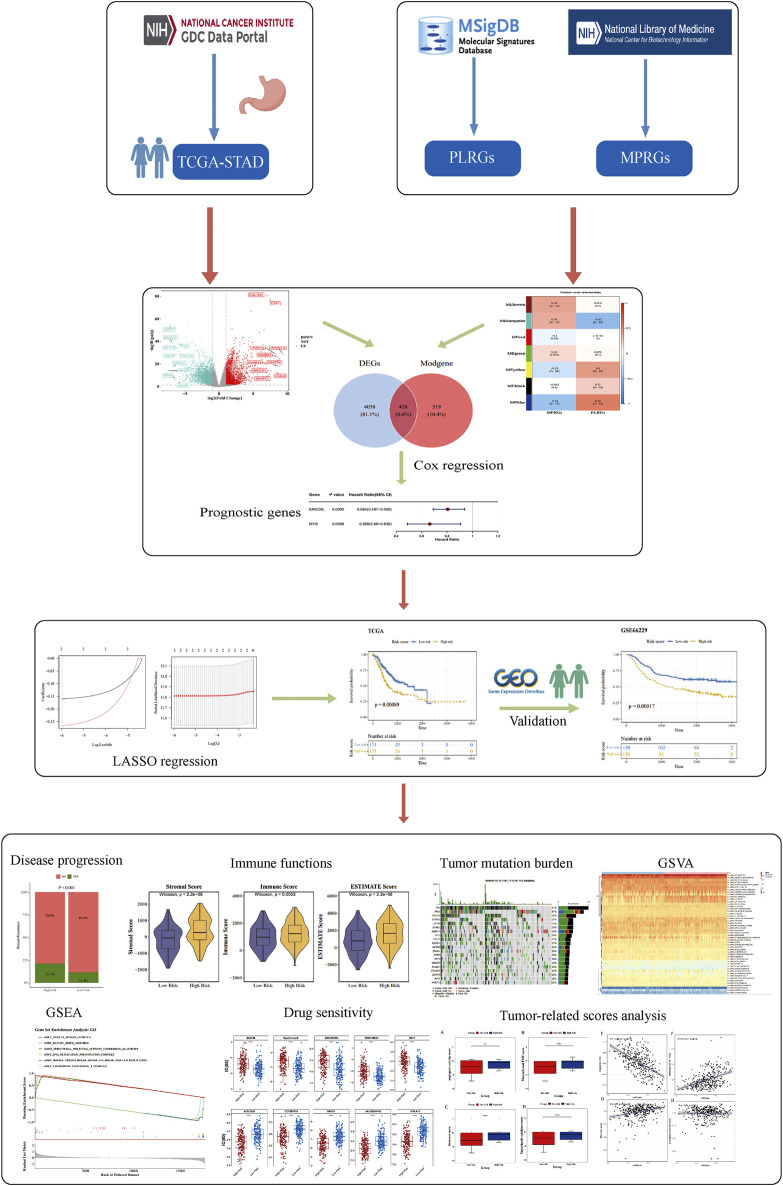

## 1 Introduction

Gastric cancer (GC) is a major global health concern, ranking as the fifth most common malignancy and the third leading cause of cancer-related deaths worldwide (Smyth et al., 2020). Despite ongoing advances in treatment, the prognosis for patients with GC remains poor, with a 5-year survival rate of under 10% (Yang et al., 2020). GC is influenced by multiple factors, including genetic predisposition, *Helicobacter pylori* infection, dietary habits (such as high salt and fat intake), chronic gastritis, and acid reflux. These factors contribute to chronic gastric mucosal damage, inflammation, gene mutations, and inactivation of tumor suppressor genes (Zen et al., 2022). Conventional chemotherapy, while a mainstay of treatment, offers limited efficacy and is often associated with significant side effects. Although targeted therapies and immunotherapies have shown promise, managing GC remains a considerable challenge (Alsina et al., 2023). Therefore, the urgent need for novel and more effective therapeutic approaches to improve patient outcomes is clear. Recent studies suggest that GC pathogenesis is primarily driven by cellular metabolic dysfunction (Zhao et al., 2022).

Lactic acid is a by-product of glucose metabolism under hypoxic conditions, providing energy and regulating cell function, gene expression and immune response (Zhang et al., 2022). Emerging evidence highlights that it is not only a nutrient, but also a signal molecule, promoting tumor growth, metastasis, drug resistance and immunosuppressionlactic acid (Apostolova and Pearce, 2022). Consequently, lactic acid is now recognized as a key signaling molecule, reshaping the tumor microenvironment (TME) rather than simply being metabolic waste (Zhou et al., 2022). Lactic acid can also modify proteins post-translationally, affecting their functionality (Wang et al., 2023). Similar to other post-translational modifications, lactylation can alter histones, induce conformational changes in chromatin, and regulate gene expression (Qu et al., 2023; Yu et al., 2024). Numerous studies have shown that lactylation plays a critical role in tumor progression and affects therapeutic responses by regulating the physiological functions of tumor cells, stem cells, and immune cells within the TME (Li et al., 2024a; Yang et al., 2024a). Lactylation-driven gene expression enhances GC progression and metastasis through the AKT-mTOR-CXCL1 pathway (Zhao et al., 2024).

Macrophages are the predominant immune cell type within the TME, exhibiting remarkable plasticity that enables them to adopt different phenotypes. This process, known as macrophage polarization, classifies macrophages into classically activated M1 and alternatively activated M2 types based on their activation state. M1 macrophages, activated by pro-inflammatory agents like LPS and IFN-γ, produce cytokines such as TNF-α, IL-6, and IL-12 to enhance inflammation and pathogen removal, primarily through the NF-κB pathway via LPS and TLR4 interaction (He et al., 2024; Yang et al., 2024b). In contrast, M2 macrophages, induced by IL-4 and IL-13, release anti-inflammatory cytokines like IL-10 and TGF-β, aiding in tissue repair, immunosuppression, and tumor growth (Chaterjee and Sur, 2023). They also secrete CHI3L1 protein, facilitating gastric and breast cancer metastasis (Chen et al., 2017). Additionally, gastric cancer cell-derived mesenchymal stem cells (GC MSCs) can induce M2 polarization, promoting gastric cancer spread and epithelial-mesenchymal transition (EMT) (Li et al., 2019).

Lactate metabolism and histone lactation in TME drive macrophage polarization and tumor progression (Gao et al., 2025). As an energy source and signal molecule, lactic acid induces M2 polarization through erk/stat3, which promotes tumor growth through anti-inflammatory and angiogenic functions (Zhang et al., 2023; Shi et al., 2022). Tumor cells release lactate, which is absorbed by tumor-associated macrophages (TAMs), enhancing M2 polarization and creating a cycle that promotes tumor growth (Chaterjee and Sur, 2023). Histone lactylation, such as lysine lactylation (KLA), influences gene expression linked to macrophage polarization (Gao et al., 2024). Lactic acid induces histone lactylation at M2-specific gene promoters (e.g., Arg-1), activating transcription and driving macrophage transition from M1 to M2 (Qu et al., 2023). Zhao et al. discovered histone lactylation modification in human breast cancer cells and macrophages from melanoma and lung tumor mouse models. This modification correlates with the carcinogenic potential of M2 macrophages (Zhang et al., 2019). Lactate metabolism and histone lactylation modification together influence macrophage polarization and tumor progression. This study aims to identify prognostic genes related to lactylation and macrophage polarization in gastric cancer to improve treatment and prognosis.

This study is the first to integrate macrophage polarization and lactylation genes, using bioinformatics and machine learning to identify prognostic genes in gastric cancer (GC). A risk model including ERCC6L, MYB was developed, considering immune infiltration and clinical recurrence data. Single-cell sequencing and functional experiments confirmed the biological function, offering new insights for personalized GC treatment.

## 2 Materials and methods

### 2.1 Data mining

Transcriptomic data for GC were obtained from The Cancer Genome Atlas (TCGA) (https://portal.gdc.cancer.gov/) and Gene Expression Omnibus (GEO) (https://www.ncbi.nlm.nih.gov/geo/). The TCGA-GC dataset, comprising 350 tumor and 31 normal gastric tissue samples, was designated as the training set. GSE66229 (GPL570 platform), which includes 300 GC samples and 100 normal samples from GEO, served as the validation set. The single-cell dataset GSE163558 (GPL24676 platform) included 3 GC and one normal sample. Thirty-five macrophage polarization-related genes (MPRGs) were retrieved from the Molecular Signatures Database (MsigDB) (https://www.gsea-msigdb.org/gsea/msigdb) (Zhao et al., 2021). Protein lactylation-related genes (PLRGs), including HDAC1, HDAC2, HDAC3, LRG1, VEGFA, IL10, EP300, SIRT1, LDHA, LDHB, KAT2A, and GCN5, were collected from published literature sources (Zhang et al., 2019; Moreno-Yruela et al., 2022; Wang et al., 2022a; Yu et al., 2021). All data were downloaded on 2 July 2024.

### 2.2 Differential expression analysis

Differentially expressed genes (DEGs) between tumor and normal samples in TCGA-GC were identified using the DESeq2 package (v1.38.0) (Love et al., 2014), with thresholds set at adjusted p-value <0.05 and |log_2_ fold change (FC)| > 1. Visualization was conducted using the ggplot2 (v3.4.4) (Gustavsson et al., 2022) and ComplexHeatmap (v2.14.0) (Gu and Hubschmann, 2022) packages for volcano plots and heatmaps, respectively.

### 2.3 Weighted gene co-expression network analysis (WGCNA)

GSVA scores for MPRGs and PLRGs were computed using the GSVA package (v1.46.0) (Hanzelmann et al., 2013). The Wilcoxon test was applied to assess score differences between tumor and normal samples (p < 0.05). Gene co-expression network analysis was performed using the WGCNA package (v1.7.1) (Langfelder and Horvath, 2008). Sample clustering via Euclidean distance enabled the exclusion of outliers. A soft-thresholding power yielding an R^2^ > 0.9 and near-zero mean connectivity was selected. Gene adjacency was calculated to estimate topological similarity, and modules were constructed using dynamic tree cutting (minimum module size = 100; merge cut height = 0.3). Modules exhibiting the strongest Spearman correlation with GSVA scores of PLRGs and MPRGs were selected. Genes within these modules (|correlation coefficient| > 0.3, p < 0.05) were defined as key module genes (Modgenes) for downstream analysis.

### 2.4 Identification and analysis of candidate genes

The VennDiagram package (v1.7.3) (Chen and Boutros, 2011) was used to identify overlapping genes between DEGs and Modgenes. Functional enrichment of overlapping genes was performed using the clusterProfiler package (v4.7.1.003) (Wu et al., 2021), with significance set at p < 0.05. Enrichment results for Gene Ontology (GO) and Kyoto Encyclopedia of Genes and Genomes (KEGG) pathways were visualized using the GOplot package (v1.0.2) (Walter et al., 2015). A high-confidence protein–protein interaction (PPI) network (interaction score = 0.9) was constructed using STRING (https://string-db.org), and visualized in Cytoscape (v3.8.2) (Liu et al., 2020) following outlier removal.

### 2.5 Construction of a risk model

Prognostic relevance of protein-interaction genes was assessed using univariate Cox regression via the survival package (v3.5–3) (Lei et al., 2023) (p < 0.01), with results visualized in a forest plot. The proportional hazards (PH) assumption was verified prior to LASSO regression analysis using the glmnet package (v4.1–4) (Li et al., 2022), which was then used to identify prognostic gene signatures. To clarify the association between prognostic genes and patient survival, samples were divided into high and low expression groups based on the optimal cutoff value of each gene in the training set. Kaplan-Meier survival curves were plotted using the survminer package (v 0.4.9) (Liu et al., 2021) to evaluate the survival differences between the two groups, with a significance threshold set at p < 0.05. These signatures were incorporated into a risk model constructed using the formula: $\text{risk score}=\sum_{i=1}^{n}\text{coef }\left({\text{gene}}_{i}\right)\times \text{expr }\left({\text{gene}}_{i}\right)$ , where β represents the regression coefficient and x denotes the expression level of the corresponding gene in each sample.

### 2.6 Evaluation and validation of prognostic risk model

In the TCGA-GC dataset, individual risk scores were computed for each patient to evaluate the prognostic model, with patients stratified into high- and low-risk groups based on the median score. Risk distribution and survival status were visualized accordingly. Kaplan–Meier survival analysis, performed using the survminer package (v0.4.9) (Liu et al., 2021), compared survival outcomes between groups. Predictive performance was assessed via time-dependent ROC curves and corresponding AUC values at 1, 2, and 3 years, generated using the survivalROC package (v1.0.3.1) (Heagerty et al., 2000). To further evaluate model accuracy, risk plots, survival status charts, K–M curves, and ROC curves were also constructed based on the risk scores in the validation set GSE66229. Additionally, the characteristic genes from the studies of Sun et al. (2024) and Yang et al. (2023) were introduced, and K-M curves and ROC curves were plotted in both the TCGA-GC dataset and the validation set GSE66229. By comparing with the model performance of published literature, the effectiveness and reliability of the prognostic model of this study were evaluated more comprehensively.

### 2.7 Independent prognostic analysis and relationship between risk score and GC recurrence

A prognostic model for overall survival in patients with GC was developed by integrating risk scores with clinical variables. Univariate Cox regression identified seven significant predictors (p < 0.01), all of which met the PH assumption (p > 0.05). These variables were subsequently subjected to multivariate Cox regression to identify independent prognostic factors. A nomogram and calibration curves were generated using the rms package (v6.5-0) (Sachs, 2017), while the timeROC package (v0.4) (Blanche et al., 2013) was used to plot ROC curves at 1, 2, and 3 years. Decision curve analysis (DCA), conducted with the ggDCA package (v1.2), further assessed clinical utility (https://www.rdocumentation.org/packages/ggDCA/versions/). Cancer recurrence was analyzed by comparing recurrence rates between high- and low-risk groups, with statistical significance determined using the chi-square test.

### 2.8 Immune infiltration analysis and mutation analysis

To investigate differences in the TME, the estimate package (v1.0.13) (Yoshihara et al., 2013) was employed to compute stromal, immune, and combined ESTIMATE scores. Intergroup differences were evaluated via the Wilcoxon test (p < 0.05). Immune cell infiltration across 22 cell types was assessed using the IOBR package (v0.99.9) (Zeng et al., 2021) and the CIBERSORT algorithm (Wang et al., 2022b). Immune cells with significantly different infiltration levels between risk groups (p < 0.05) were visualized using box plots. Correlations between prognostic gene expression and differential immune cell populations were examined using Spearman correlation analysis (|cor| > 0.30, p < 0.05) via the psych package (v3.4.4) (Correction to Lancet Psych, 2022, 2023).

Genomic alterations across risk groups were profiled using the maftools package (v2.14.0) (Mayakonda et al., 2018). Mutation data were visualized with waterfall plots depicting the top 20 most frequently mutated genes in each group.

### 2.9 Gene set enrichment analysis (GSEA) and gene set variation analysis (GSVA)

To explore gene expression differences between risk groups, differential expression analysis was conducted using DESeq2 (v1.38.0), with genes ranked by descending log_2_ FC. Gene set enrichment analysis (GSEA) was performed via clusterProfiler, using the c5. go.v7.4. symbols gene set from MSigDB (https://www.gsea-msigdb.org/gsea/msigdb), applying thresholds of |NES| > 1 and p < 0.05. Hallmark pathway activity scores for each sample were computed using the GSVA package (v1.46.0) (Hanzelmann et al., 2013), with intergroup comparisons evaluated using the Wilcoxon test (p < 0.05). The psych package (v3.4.4) was used for Spearman correlation analysis to link prognostic gene expression to enriched GSVA pathways (|cor| > 0.30, p < 0.05).

### 2.10 Drug sensitivity analysis

To further investigate chemotherapeutic responses across risk groups, the pRRophetic package (v0.5) (Geeleher et al., 2014) was employed to estimate the half-maximal inhibitory concentration (IC_50_) values for 138 commonly used chemotherapeutic and targeted agents in TCGA-GC patient samples. In the high-risk group, the five compounds exhibiting the most elevated IC_50_ values and the five with the most reduced IC_50_ values were identified. Group-wise differences were visualized using box plots. Additionally, the ComplexHeatmap package (v2.14.0) was used to depict the expression patterns of the top 20 DEGs between risk groups. Chemical structures of the aforementioned 10 compounds were retrieved from the PubChem database (https://pubchem.ncbi.nlm.nih.gov/) for visualization.

### 2.11 Tumor-related scores analysis

Scores representing angiogenesis, epithelial-mesenchymal transition (EMT), tumorigenic cytokine activity, and stemness were obtained from previously published research (Zhang et al., 2024) and calculated using single-sample gene set enrichment analysis (ssGSEA). Group differences were evaluated via the Wilcoxon test, and associations between these indicators and the risk score were assessed using Spearman correlation analysis.

### 2.12 Chromosome localization, construction of gene-gene interaction (GGI) network, and the expression of prognostic genes

Genomic localization of prognostic genes was analyzed with the RCircos package (v1.2.2) (Zhang et al., 2013). A gene–gene interaction (GGI) network was constructed to illustrate interactions among prognostic genes and their associated partners. Expression levels of prognostic genes were compared between tumor and normal tissues in both the TCGA-GC and GSE66229 datasets using the Wilcoxon test (p < 0.05), providing robust cross-cohort validation.

### 2.13 Single-cell RNA sequencing analysis

In order to explore the expression of key genes at the single-cell level, based on the GSE163558 single-cell sequencing data set, Seurat package (v 5.2.99.9006) (Butler et al., 2018) was used to integrate and strictly filter the original data: (1) Cells with the number of genes <200 or the proportion of mitochondrial genes >10% were removed; (2) Genes with the number of covered cells <3 were removed; (3) The capping method was applied to remove outliers, and abnormal cells with the gene count per cell between 200 and 3,000 and total counts ≥10,000 were retained; (4) Cells with the proportion of mitochondrial genes >10% were removed. After data normalization, the “FindVariableFeatures” function was used to extract 2,000 highly variable genes with the largest variation to reduce the amount of calculation. PCA analysis was performed on different samples, and they were sorted according to the variance percentage of principal components; the JackStrawPlot function was used to compare the *p*-value distribution of each PC through the permutation test method based on the zero distribution, and the first 20 principal components (dims = 20) with p < 0.05 were selected for subsequent analysis. The FindNeighbors and FindClusters functions in the Seurat package (v 5.2.99.9006) (Butler et al., 2018) were used to perform unsupervised clustering analysis on cells, with the resolution set to 0.4, and the UMAP clustering method was used to visualize the results. In order to further explore the cell types specifically contained in each cell cluster, marker genes were obtained from the reference (Jin et al., 2021) to annotate the cell clusters, cells in different groups were clustered and the expression of core genes was presented, and cells with significant differential expression were regarded as key cells.

In order to understand the biological functions in which the annotated cells were involved, ReactomeGSA package (v 1.16.1) (Griss et al., 2020) was used to explore the function enrichment of each cell in GC samples and control samples and explore the biological pathways. Subsequently, based on the single-cell data, the R package CellChat package (2.2.0) (Jiang et al., 2022) was used to analyze the expression and pairing of cell receptors and ligands and infer the cell-cell interactions. Finally, based on the key cell clusters, re-dimensionality reduction and clustering were carried out (dim = 20, resolution = 0.4), and the Monocle package (2.30.1) (Cao et al., 2019) was used to perform pseudo-time-series analysis on the key cells and present the expression of key genes at different time stages.

### 2.14 Cell culture and quantitative real-time PCR (qRT-PCR) assay

Human GC cell lines HGC-27, AGS and normal gastricepithelial cell line GES-1 were obtained from Procell Life Sciences and Suzhou Hysigen Biotechnology, respectively. HGC-27 and AGS were cultured in RPMI 1640 medium (Gibco). GES-1 was cultured in DMEM with high glucose medium (Gibco). The medium all contained 10% fetal bovine serum (Procell) and 1% penicillin-streptomycin (Solarbio).

RNA was extracted using Trizol (Takara), and cDNA was synthesized with the RevertAid RT Kit (Thermo Scientific). Following the instructions, 5 μg of RNA were converted into 10 μL of cDNA. RT-qPCR began with a 15-min pre-denaturation at 95°C, followed by 30 s at 95°C and 60 cycles at 60°C. The dissociation and dissolution curves were both conducted at 95°C. The BIO-RAD CFX96 system performed qRT-PCR, using β-actin as a reference. Primer sequences are listed in Supplementary Table S1. RNA expression was analyzed using the 2^−ΔΔCT^ method.

### 2.15 Tumor samples collection

From September 2022 to April 2024, formalin-fixed paraffin-embedded tissue samples were collected from 48 GC and adjacent non-tumor specimens at the First Affiliated Hospital of Kunming Medical University. All patients underwent radical gastrectomy and had received no prior anticancer treatments such as targeted therapy or radiotherapy. Complete clinical data were available for all cases.

### 2.16 Immunohistochemistry (IHC) analysis

IHC was performed using anti-ERCC6L (Proteintech, #15688-1-AP), anti-L-lactyl lysine (PTM-1401RM), and anti-MYB (Bioss, #bs-5978R) antibodies, following standard protocols (Pan et al., 2021). Two independent pathologists, blinded to clinical outcomes, scored the IHC results. Staining intensity was graded as 0 (negative), 1 (weak), 2 (moderate), or 3 (strong), while the proportion of positively stained cells was scored as 0 (1%–5%), 1 (6%–25%), 2 (26%–50%), 3 (51%–75%), or 4 (76%–100%). The final IHC score was calculated as the product of intensity and proportion scores.

### 2.17 Statistical analysis

All statistical analyses were conducted using R software (v4.2.3). Group differences were assessed using the Wilcoxon test, while RT-qPCR results were evaluated via ANOVA. A p-value <0.05 was considered statistically significant.

## 3 Results

### 3.1 Identification of candidate genes

**FIGURE 1:**
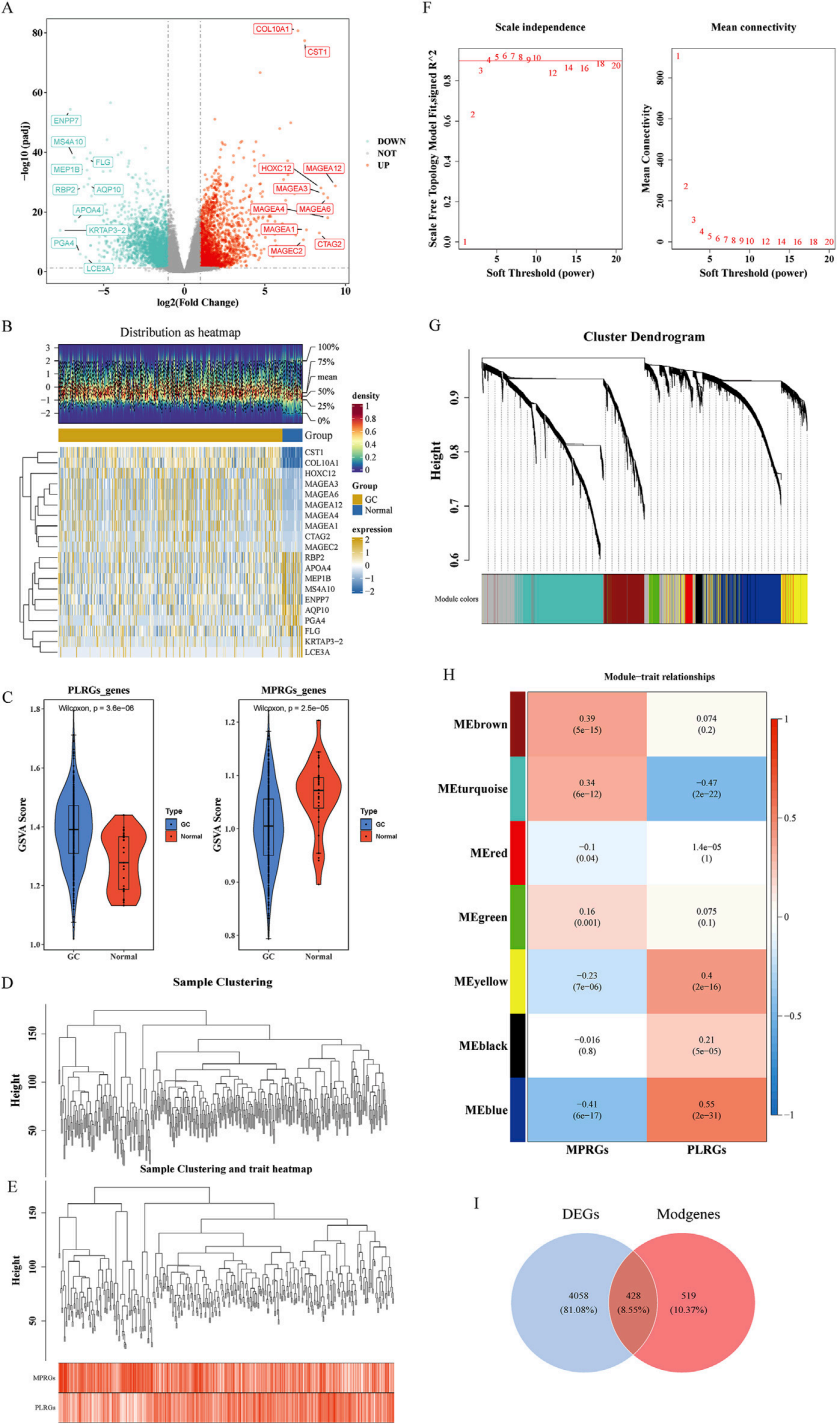
Candidate gene identification. **(A)** Volcano plot illustrating the differential expression of DEGs. Each point in the plot represented a gene, where gray points indicated genes with no significant expression, blue points represented significantly downregulated genes, and red points denoted significantly upregulated genes; the top 10 upregulated and downregulated genes were labeled. **(B)** Heatmap displaying expression profiles of DEGs. The lower part showed the expression heatmap of the top 10 upregulated and downregulated genes across samples, with the horizontal axis representing samples and the vertical axis representing genes. The blue color above indicated normal samples, and the yellow color represented GC patient samples; within the plot, yellow indicated high gene expression, and blue indicated low gene expression. The upper part was a density heatmap of the expression levels of the top 10 upregulated and downregulated genes in samples, displaying lines for the five quantiles and the mean. **(C)** Violin plot comparing PLRGs and MPRGs between groups. **(D, E)** Samples clustered into two distinct subtypes with significant divergence; all clusters were retained for downstream analysis. **(F)** Determination of optimal soft-thresholding power. **(G)** Gene dendrogram with corresponding module assignment. The upper part was a hierarchical clustering dendrogram of genes, and the lower part showed gene modules, i.e., network modules. **(H)** Correlation heatmap between modules and phenotypes. The color blocks on the far left represented modules, and the color bar on the far right indicated the correlation range. In the central heatmap, darker colors indicated higher correlation, with red representing positive correlation and blue representing negative correlation; the numbers in each cell indicated the correlation coefficient and significance. **(I)** Venn diagram indicating the intersection and quantity of candidate genes.

Differential expression analysis identified 4,486 DEGs between GC and normal samples, including 2,117 upregulated and 2,369 downregulated genes in GC (p < 0.05) (Figures 1A,B). PLRGs scores were elevated, while MPRGs scores were reduced in the GC cohort (Figure 1C). WGCNA, performed using a soft threshold of 6, detected no outliers and constructed a hierarchical clustering of MPRGs and PLRGs scores (Figures 1D–F), revealing seven gene modules (Figure 1G). Among these, the MEblue module demonstrated the strongest correlation with PLRGs (cor = 0.55, p < 0.05) and MPRGs (cor = −0.491, p < 0.05), comprising 947 module-related genes (Modgenes) (Figure 1H). Intersecting the 4,486 DEGs with these Modgenes yielded 428 candidate genes (Figure 1I).

### 3.2 Functional analysis of candidate genes

**FIGURE 2:**
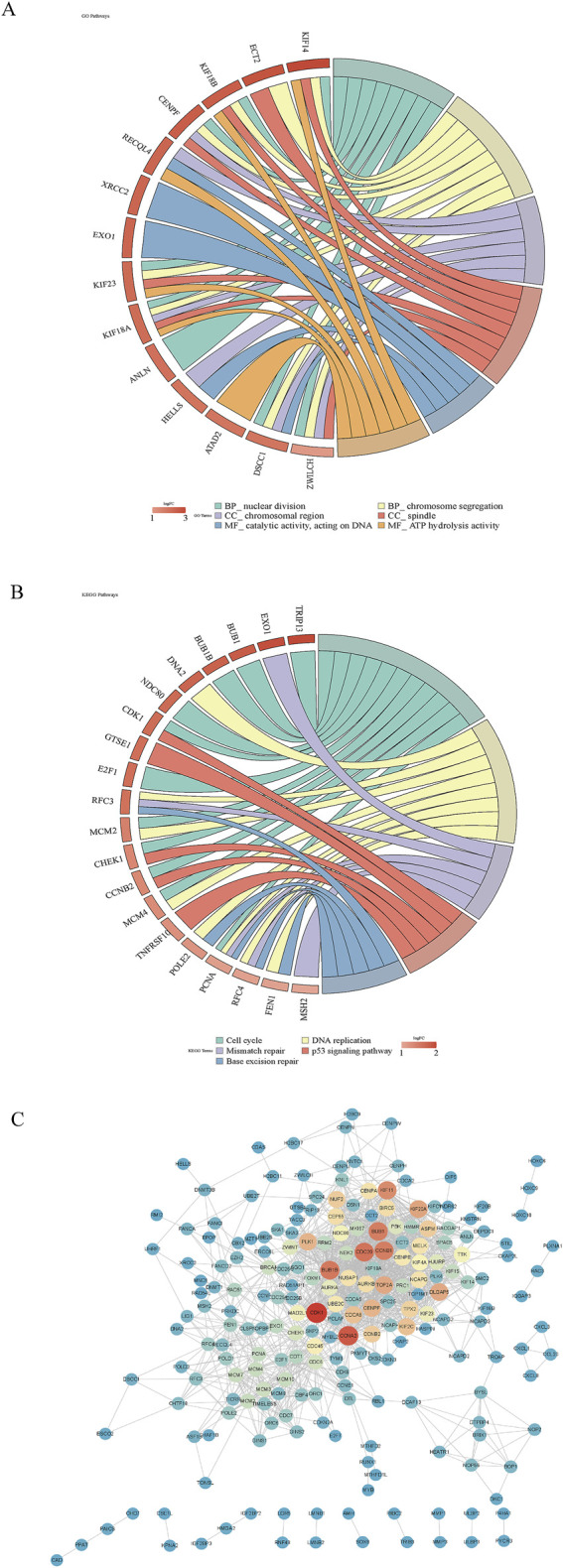
Functional enrichment analysis of candidate genes. **(A, B)** GO and KEGG pathway analyses of candidate genes. Genes were on the left, and pathways were on the right, with pathway names displayed below. **(C)** PPI network construction to explore gene–gene interactions among candidates. The redder the color was, the higher the connectivity was.

Functional enrichment analysis highlighted key biological processes, pathways, and molecular interactions implicated in disease mechanisms. Analysis of the candidate genes revealed 430 GO terms—341 biological processes, 63 cellular components, and 26 molecular functions—including nuclear division, chromosomal region, and ATP hydrolysis activity (Figure 2A). Additionally, 199 KEGG pathways were enriched, notably the cell cycle (Figure 2B). The PPI network constructed from these candidates comprised 215 nodes and 1,432 edges, resulting in 215 high-confidence protein interaction genes after filtering (Figure 2C).

### 3.3 Construction, assessment, and validation of prognostic risk model

**FIGURE 3:**
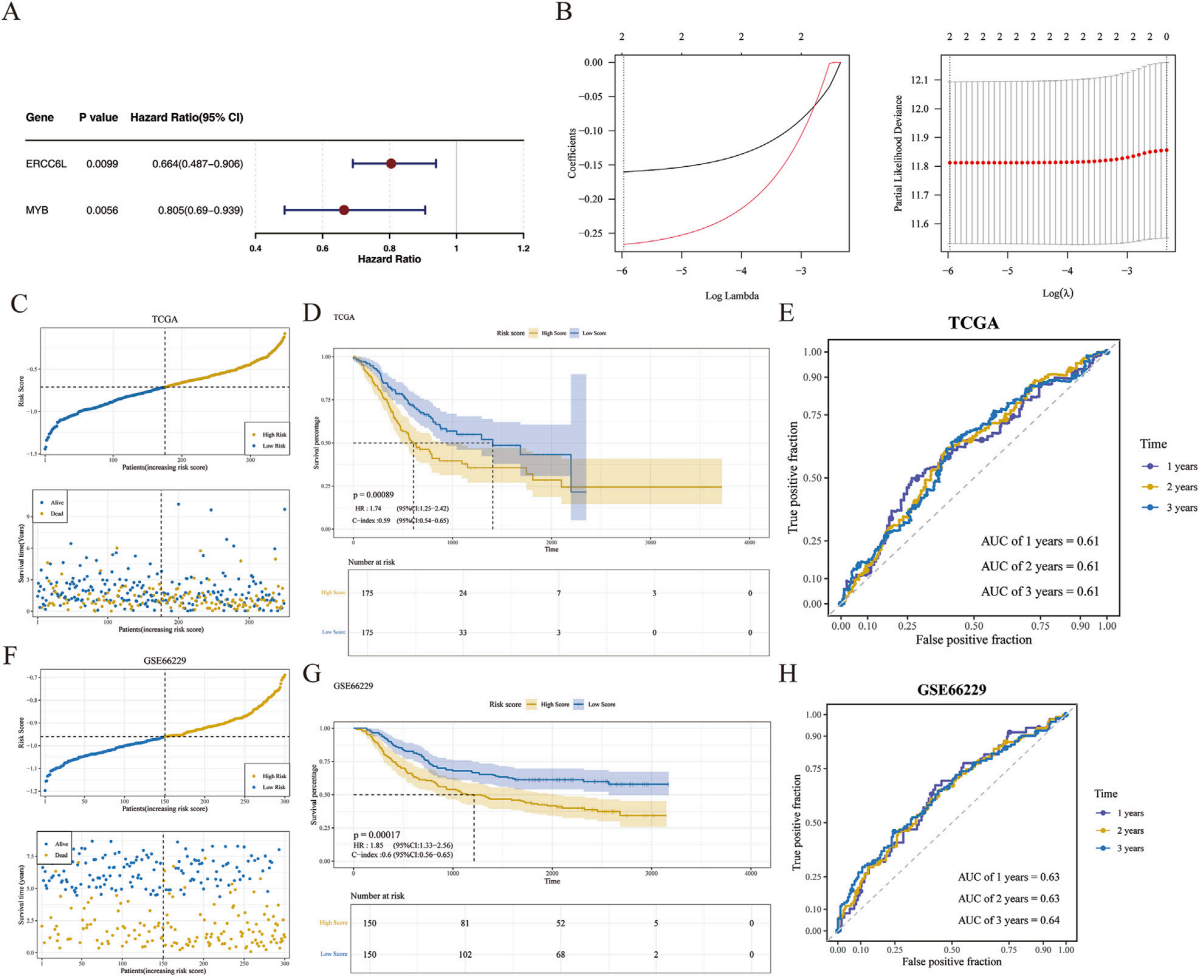
Risk model development and validation. **(A)** Forest plot of prognostic genes, with 95% confidence intervals for hazard ratios; genes with HR > 1 and p < 0.05 identified as adverse prognostic markers. **(B)** LASSO regression used to construct a two-gene signature at the optimal λ value. Left panel: LASSO coefficient spectrum plot. The abscissa was the logarithm of lambdas, and the ordinate was the variable coefficient, with each line representing a gene. As lambdas increased, the variable coefficients of the genes approached zero. When the optimal lambda was reached, variables with coefficients equal to zero were excluded. Right panel: Tenfold cross‐validation for adjusting parameters in the LASSO analysis. The abscissa was the logarithm of lambdas, and the ordinate was the model error. **(C, F)** The risk score of the training and verification sets, respectively. In the upper graph, the abscissa represented patients, and the ordinate represented risk scores. The risk scores of patients increased from left to right, with yellow dots indicating high‐risk patients and blue dots indicating low-risk patients. In the lower graph, the abscissa represented patients, and the ordinate represented survival time, with yellow dots indicating deceased patients and blue dots indicating surviving patients. **(D, G)** Kaplan–Meier survival curves comparing overall survival between groups in the TCGA and GSE66229 cohorts. **(E, H)** Receiver operating characteristic (ROC) curves assessing the discriminatory performance of the model in both datasets.

A prognostic risk model was developed to stratify patients with GC based on multi-omics profiles, facilitating precision medicine through targeted interventions in high-risk groups. Univariate Cox regression analysis identified two prognostic genes, MYB and ERCC6L, both satisfying the PH assumption and retaining significance in LASSO regression (Figures 3A,B). Further analysis showed that the optimal expression cutoff value for ERCC6L was 1.256032, based on which the samples were divided into a low-expression group (164 cases) and a high-expression group (186 cases). The optimal cutoff value for MYB was 2.973839, corresponding to a low-expression group (255 cases) and a high-expression group (95 cases). Kaplan-Meier survival curves indicated that patients with high expression of ERCC6L and MYB had significantly longer survival than those with low expression (P < 0.05) (Supplementary Figure S1), suggesting that high expression of these two genes may be associated with improved prognosis in gastric cancer patients. The risk score was calculated as: Risk score = (−0.1602052) × MYB expression + (−0.2663049) × ERCC6L expression. Samples were stratified by median risk score (−0.7115388) into high/low-risk groups (175 cases each) (Figure 3C), with the high-risk group showing poorer survival (p < 0.05) (Figure 3D). The model yielded AUC values of 0.61 at 1, 2, and 3 years, indicating moderate predictive performance (Figure 3E). In the validation cohort, 150 GC samples were similarly stratified (Figure 3F), with survival trends consistent with the training set (Figure 3G) and AUCs of 0.63, 0.63, and 0.64 at 1, 2, and 3 years, respectively, supporting the model’s robustness (Figure 3H).In the model comparison analysis (Supplementary Table S2), Sun et al.'s three-gene model (COL4A1, SLC16A7, IRAK1) and the two-gene model developed in this study exhibited comparable predictive performance. In the TCGA-GC training cohort (Supplementary Figures S2A–S2B), Sun’s model achieved a C-index of 0.60, with 1, 2, and 3 years AUC values of 0.65, 0.62, and 0.62, respectively. In the GSE66229 (Supplementary Figures S2C–S2D), Sun’s model maintained a C-index of 0.60, accompanied by 1, 2, and 3 years AUC values of 0.62, 0.61, and 0.63. Although Yang et al.’s lactylation scoring model was not directly compared due to methodological differences, the comparative analysis between Sun’s model and our model revealed minimal differences in C-index and time-dependent AUC values. These findings indicated that while maintaining similar predictive efficacy, our two-gene model potentially offered a more streamlined clinical application advantage by utilizing fewer genes.

### 3.4 Clinical characteristics and the assessment of GC recurrence analysis

**FIGURE 4:**
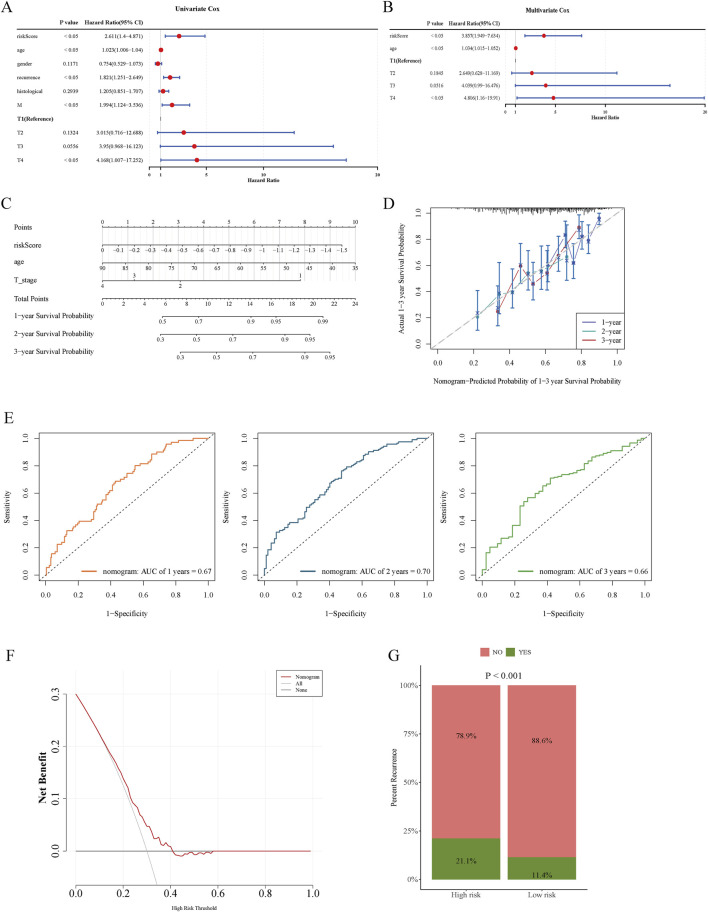
Forest plots from univariate and multivariate Cox regression analyses, together with nomograms and calibration curves, generated using clinical parameters and risk scores. **(A)** Forest plot from univariate Cox regression analysis. **(B)** Forest plot from multivariate Cox regression analysis. **(C)** Nomogram developed using the TCGA cohort. **(D)** Calibration curves for 1-, 2-, and 3-year survival probabilities. **(E)** Time-dependent ROC curves at 1, 2, and 3 years evaluating model predictive accuracy. **(F)** DCA curves illustrating the clinical utility of the model. **(G)** Recurrence status comparison between high- and low-risk patient groups.

Survival analysis using Cox regression was employed to identify prognostic determinants in GC. Risk score, age, recurrence status, and M and T stages demonstrated significant associations with overall survival (Figure 4A). However, recurrence and M stage violated the PH assumption, limiting their utility in multivariate modeling. After adjusting for confounders, multivariate Cox analysis identified risk score, age, and T stage as independent predictors of overall survival (Figure 4B). A nomogram incorporating these variables was constructed to evaluate their cumulative impact on prognosis (Figure 4C), with calibration curves aligning closely with the diagonal line, indicating high predictive accuracy (Figure 4D). The nomogram achieved AUC values of 0.67, 0.70, and 0.66 at 1, 2, and 3 years, respectively, demonstrating strong predictive performance (Figure 4E). DCA further confirmed that the nomogram provided greater net clinical benefit than either the treat-all or treat-none strategies (Figure 4F). Notably, cancer recurrence rates were significantly higher in the high-risk group relative to the low-risk group (Figure 4G).

### 3.5 Immune infiltration and mutation analysis

**FIGURE 5:**
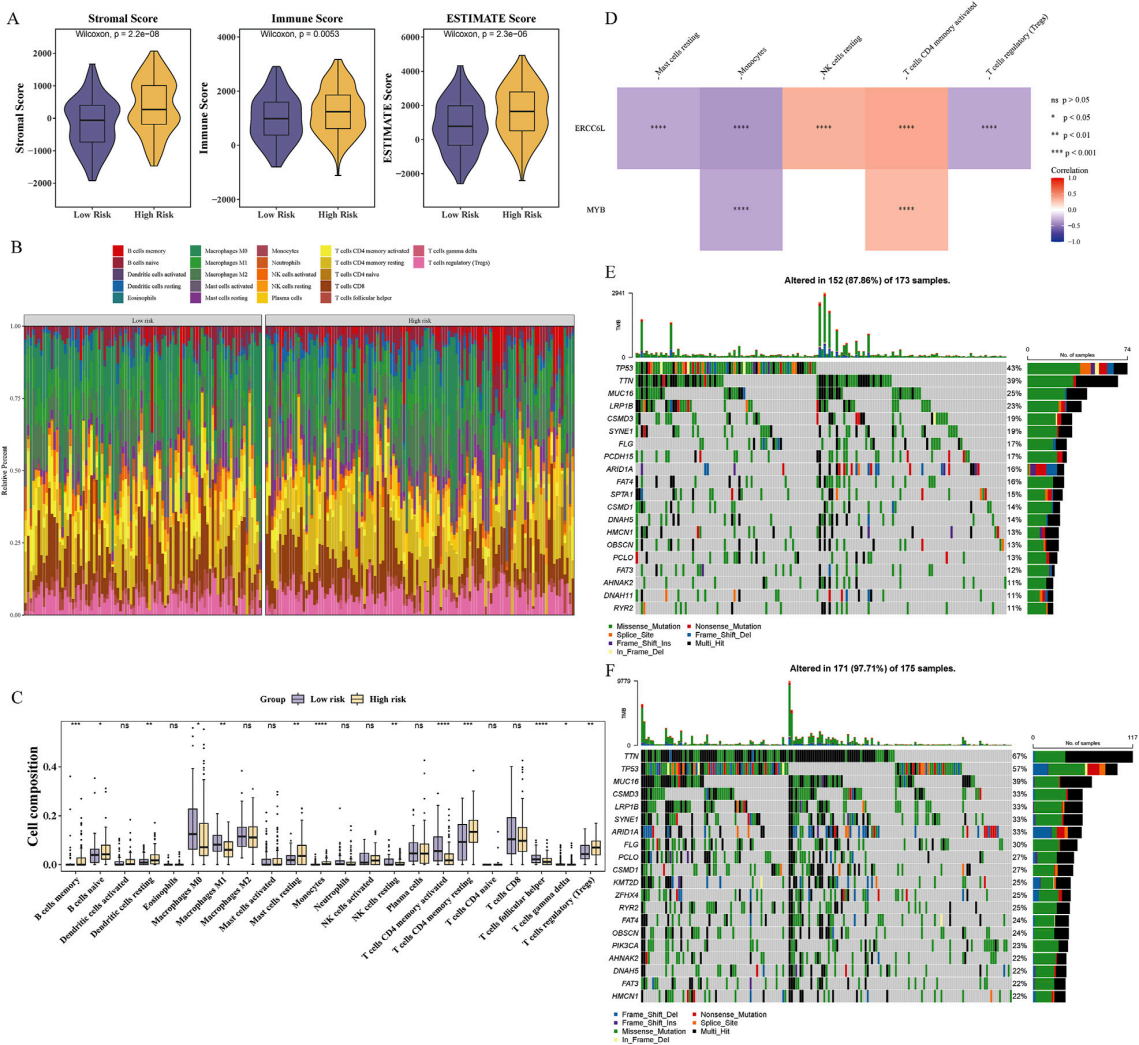
Immune infiltration and mutation differences between groups. **(A)** The immunescore of high-risk group was significantly lower than that of low-risk group. **(B)** Immune cell infiltration in GC patient samples. **(C)** Differential analysis of immune cells. “ns” represented no significance, “*” represented P < 0.05, and “**” represented *p* < 0.01, “***” represented *p* < 0.001, and “****” represented *p* < 0.0001. **(D)** Correlations between prognostic genes and differentially infiltrated immune cells. Red indicated positive correlation, and blue indicated negative correlation. “****” represented *p* < 0.0001. **(E, F)** Waterfall plots illustrating somatic mutation distributions in high-risk **(E)** and low-risk **(F)** subgroups.

Immune infiltration analysis underscored its pivotal role in GC progression and prognosis, offering insights into immune-related therapeutic opportunities. The high-risk group exhibited significantly elevated immune, stromal, and estimate scores, suggesting increased immune cell infiltration and TME complexity (Figure 5A). The composition of 22 immune cell types was profiled across risk groups, revealing distinct immunological landscapes (Figure 5B). Significant differences in immune cell proportions (p < 0.05) were observed between groups, with 13 immune cell types showing differential abundance, including regulatory T cells (Tregs) (Figure 5C). Several immune cell types displayed strong associations with prognostic genes. ERCC6L expression was negatively correlated with Tregs and positively correlated with resting NK cells (|cor| > 0.3, p < 0.001) (Figure 5D), while MYB showed a negative correlation with monocytes (cor = −0.323, p < 0.001) and a positive correlation with activated CD4 memory T cells (cor = 0.302, p < 0.001) (Supplementary Table S3). Gene mutations are critical drivers of GC pathogenesis, providing mechanistic insights and informing the development of targeted and personalized therapeutic strategies. TTN, TP53, and MUC16 represent the most frequently mutated genes across both risk groups, with missense mutations predominating (Figures 5E,F). Notably, the high-risk group exhibited significantly elevated immune and stromal scores, accompanied by distinct immune cell infiltration patterns and notable associations between prognostic gene expression and specific immune cell subsets.

### 3.6 GSEA and GSVA

**FIGURE 6:**
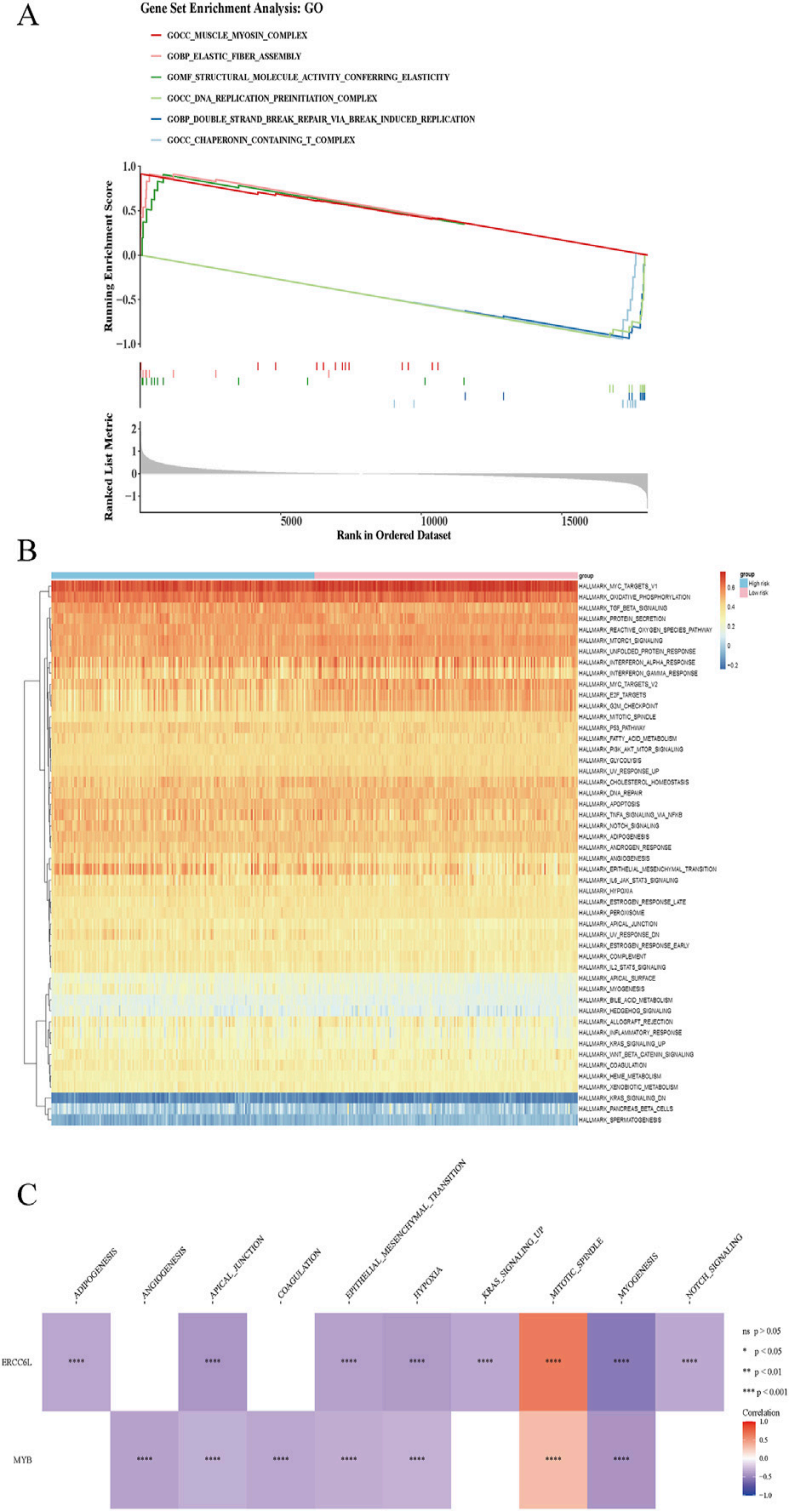
GSEA and GSVA in high- and low-risk patients. **(A)** GSEA enrichment analysis in high/low risk group. At the top was a line chart of enrichment scores, where each line represented a pathway, and the peak of each line was the enrichment score of that pathway. The genes before the peak were the core genes under the gene set of this pathway. A peak in the upper left corner indicated that the core genes were mainly upregulated genes based on the differential analysis between high and low risk groups, while a peak in the lower right corner indicated that the core genes were mainly downregulated genes based on the differential analysis between high and low risk groups. The second part marked the genes located in the gene set with lines. The third part was the distribution map of rank values for all genes. **(B)** GSVA pathway results in high/low risk group. The abscissa represented samples, and the ordinate represented signaling pathways. The blue color above indicated the high-risk group, and the pink color represented the low-risk group. **(C)** Correlation analysis revealed associations between prognostic gene expression and pathway-level alterations. Red indicated positive correlation, and blue indicated negative correlation. “****” represented *p* < 0.0001.

GSEA and GSVA are analytical strategies for interrogating gene expression data, with GSEA identifying statistically enriched pathways among differentially expressed genes, and GSVA quantifying gene set activity across individual samples. These approaches reveal intricate transcriptional signatures, deepening mechanistic insights into disease biology and supporting the development of more precise diagnostic and therapeutic interventions. GSEA revealed 118 enriched pathways, with the top-ranked including the muscle myosin complex, elastic fiber assembly, and structural molecule activity associated with elasticity. In contrast, pathways such as the DNA replication preinitiation complex, chaperonin-containing T-complex, and double-strand break repair via break-induced replication exhibited the lowest enrichment scores (p < 0.05) (Figure 6A). GSVA identified 18 significantly altered Hallmark pathways between groups, including adipogenesis, allograft rejection, and angiogenesis (Figure 6B). Correlation analysis showed strong positive associations between ERCC6L (cor = 0.632, *p* < 0.05) and MYB (cor = 0.314, *p* < 0.05) with the mitotic spindle pathway, alongside marked negative correlations with other signaling cascades (Figure 6C; Supplementary Table S4).

### 3.7 Drug sensitivity

**FIGURE 7:**
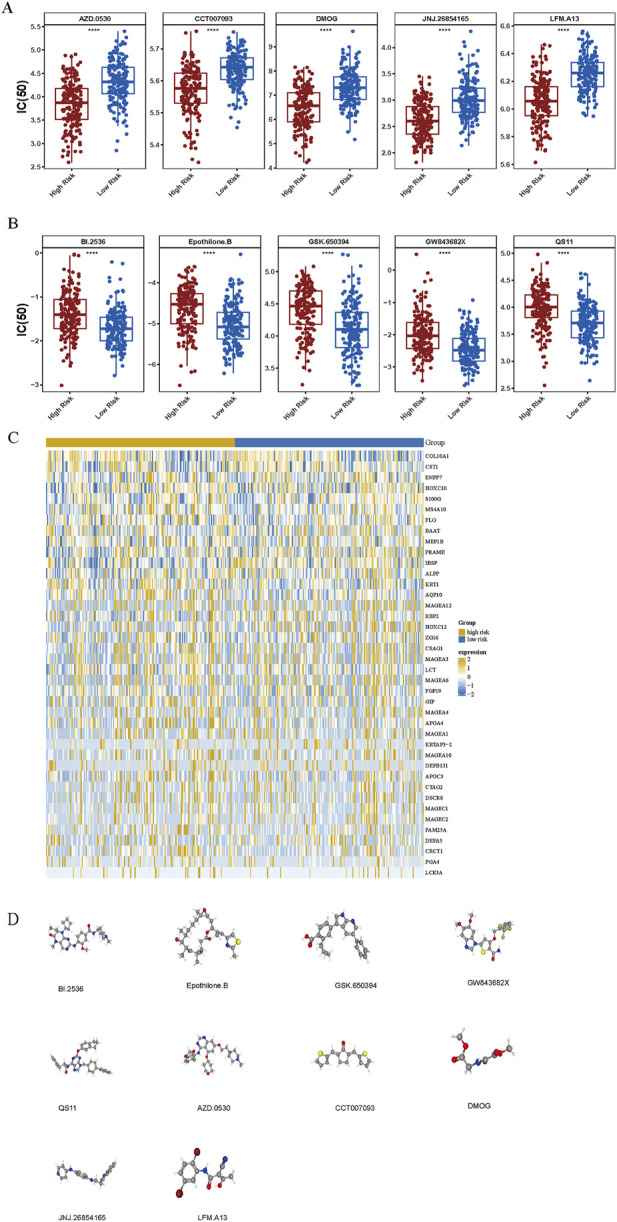
Chemotherapeutic sensitivity across risk groups. **(A, B)** Comparative analysis of chemotherapeutic response demonstrated differential drug sensitivity profiles between high- and low-risk groups. “****” represented *p* < 0.0001. **(C)** Differential gene expression analysis between the two risk categories. **(D)** Three-dimensional molecular structures of eight candidate therapeutic compounds identified via the cMap database.

Drug sensitivity testing enables individualized cancer therapy by aligning pharmacological responses with tumor-specific genetic profiles, thereby enhancing therapeutic efficacy and safety while reducing adverse effects. In GC, small-molecule agents are of particular therapeutic significance. A comparative analysis of IC_50_ values for 138 compounds in two patients with GC identified 92 drugs with statistically significant differences (*p* < 0.05) (Supplementary Table S5). Among these, 43 compounds exhibited elevated IC_50_ values and 49 demonstrated reduced IC_50_ values in the high-risk cohort. Notably, AZD.0530, CCT007093, DMOG, JNJ.26854165, and LFM. A13 showed decreased IC_50_ values, whereas BI.2536, Epothilone. B, GSK.650394, GW843682X, and QS11 displayed increased IC_50_ values in this group (Figures 7A,B). Expression profiles of the top 40 DEGs were visualized using a heatmap across risk stratifications (Figure 7C). Additionally, chemical structures for 10 selected compounds were retrieved from PubChem (Figure 7D).

### 3.8 Tumor-related scores analysis

**FIGURE 8:**
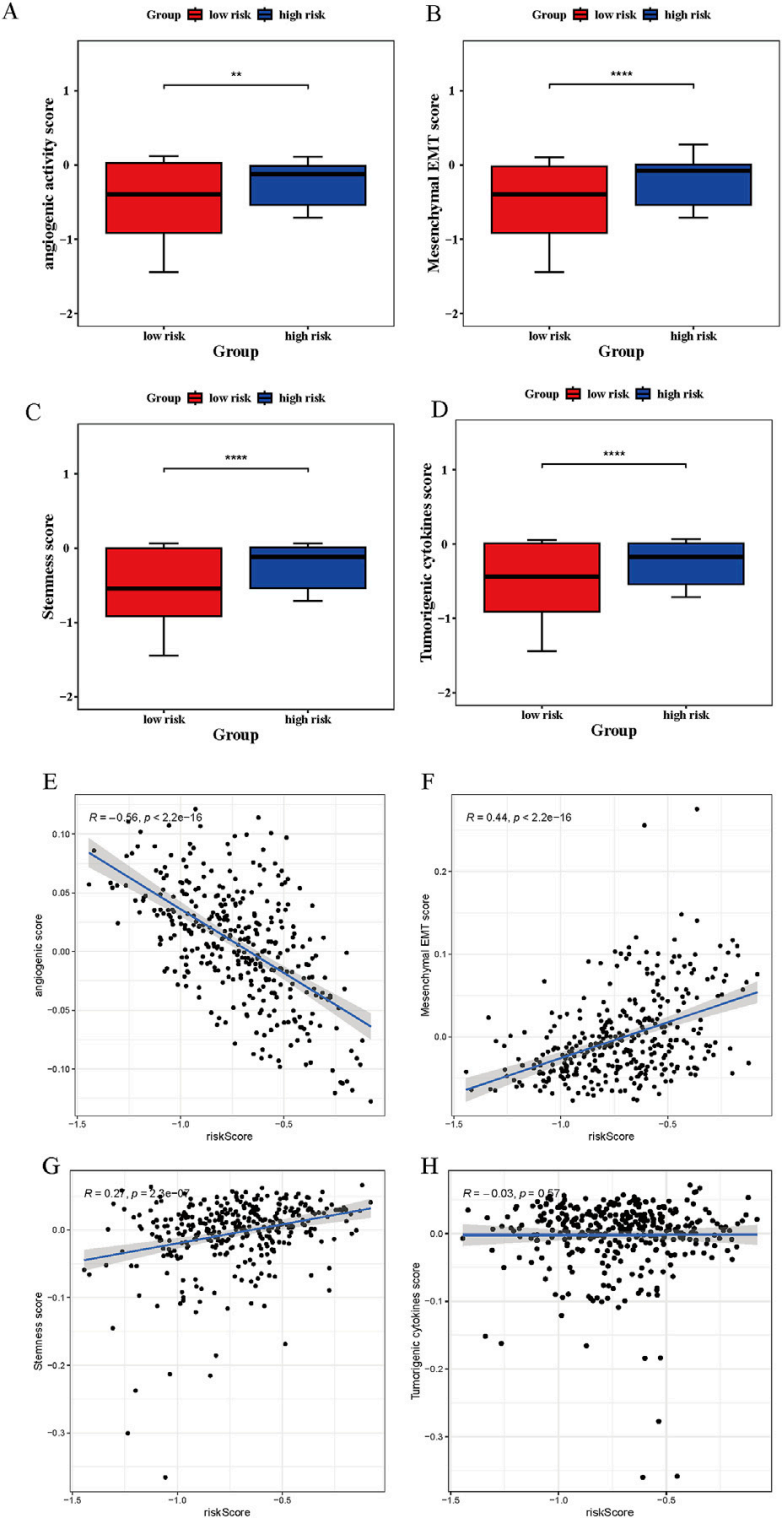
Tumor-related scores analysis. **(A–D)** Significant disparities in angiogenesis, mesenchymal transition (EMT), tumorigenic cytokine profiles, and stemness signatures observed between high- and low-risk patients. “**” represented *p* < 0.01, and “****” represented *p* < 0.0001. **(E–H)** Correlation assessments indicated strong associations between risk scores and tumor-promoting molecular features, including angiogenic activity, EMT, cytokine production, and stemness potential.

The functional analysis focused on four tumor-associated biological processes in relation to risk status. Angiogenic activity, mesenchymal EMT, stemness indices, and tumorigenic cytokine signatures were markedly elevated in the high-risk group (Figures 8A–D). Angiogenic activity exhibited a significant inverse association with the risk score (cor = −0.56, p < 0.05), whereas mesenchymal EMT was positively correlated (cor = 0.44, p < 0.05) (Figures 8E,F). In contrast, stemness indices and tumorigenic cytokine levels demonstrated no significant correlation with risk scores (|cor| < 0.3, p < 0.05) (Figures 8G,H).

### 3.9 Chromosome localization, GGI network, and expression validation of prognostic genes

**FIGURE 9:**
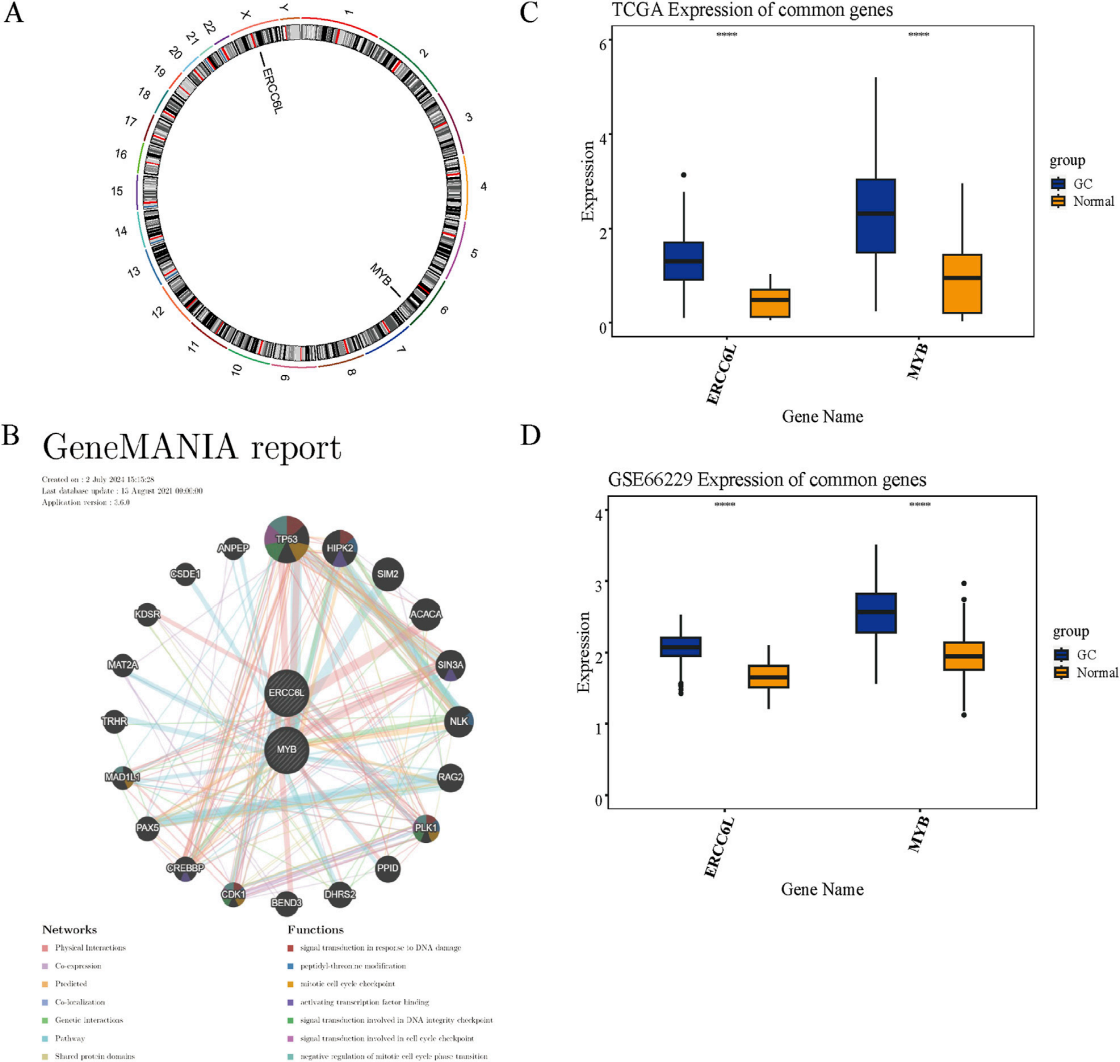
Chromosome mapping, GGI network, and expression validation of prognostic genes. **(A)** Genomic mapping illustrating chromosomal distribution of prognostic genes. **(B)** GGI network delineating interrelationships among prognostic candidates. **(C, D)** Expression levels of these genes assessed in the TCGA-GC cohort **(C)** and validated in the GSE66229 dataset **(D)**. “****” represented *p* < 0.0001.

Chromosomal localization analysis facilitates accurate gene mapping and detection of genomic clusters and abnormalities and offers critical insights into evolutionary trajectories and gene regulation. This approach supports the identification of therapeutic targets, the advancement of personalized medicine, and the discovery of diagnostic or prognostic biomarkers. ERCC6L is located on the X chromosome, while MYB is mapped to chromosome 6 (Figure 9A). Analysis of GGI networks uncovers functional associations that illuminate key regulatory pathways and disease mechanisms. The GGI network identified functional interactions among prognostic genes, including TP53, HIPK2, SIM2, ACACA, SIN3A, NLK, RAG2, PLK1, PPID, DHRS2, BEND3, CDK1, CREBBP, PAX5, MAD1L1, TRHR, MAT2A, KDSR, CSDE1, and ANPEP, primarily implicating them in the mitotic cell cycle checkpoint (Figure 9B). Differential expression analysis revealed consistent overexpression of these genes in GC samples relative to normal tissues in both TCGA and GSE66229 datasets (Figures 9C,D).

### 3.10 The expression of MYB and ERCC6L in key cell T cells

**FIGURE 10:**
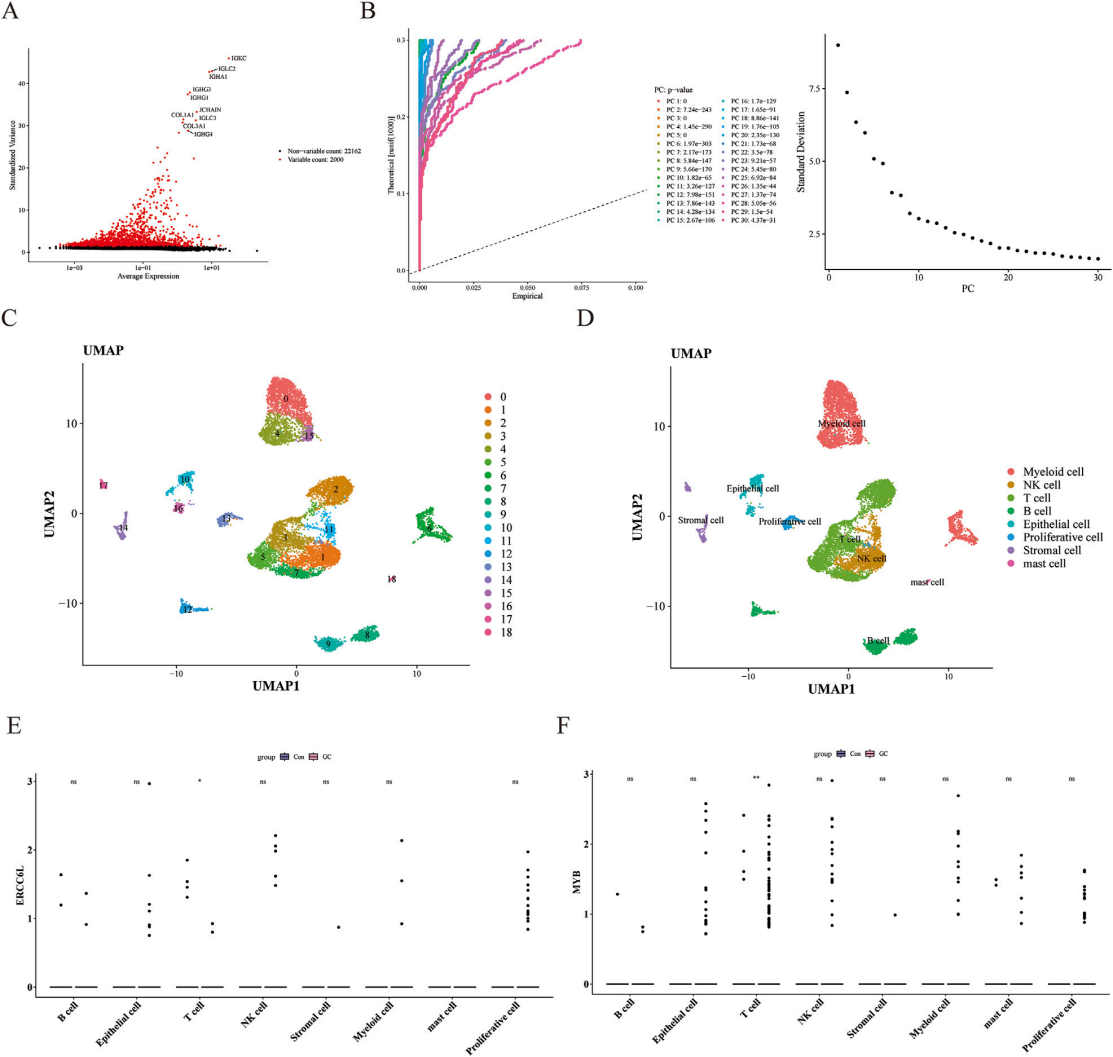
Quality control of single-cell data and cell annotation to identify key genes. **(A)** Screening of highly variable genes. Red dots represented highly variable genes, and the names of the top 10 genes were labeled. **(B)** Result graph of principal component analysis. The left side was the JackStraw graph, and the right side was the principal component inflection point graph. **(C)** Cell UMAP clustering graph. Different colors represented different clusters. **(D)** Cell-annotation UMAP graph. Each color represented a type of cell. **(E, F**) Expression of core genes in different cells. “ns” represented no significance, “*” represented *p* < 0.05, and “**” represented *p* < 0.01.

During the single-cell RNA sequencing analysis, quality control was carried out on the GSE163558 data set. After data filtering, the number of cells was 10,418, and the number of genes was 24,162 (Supplementary Figure S3A). After downscaling, the top 2,000 highly variable genes were selected (Figure 10A). PCA determined the top 20 components for subsequent analysis (Figure 10B; Supplementary Figure S3B). Through UMAP clustering, the filtered cells were divided into 19 clusters (Figure 10C) and annotated as eight cell types, namely Stromal cell, Epithelial cell, T cell, Mast cell, B cells, Proliferative cell, NK cell, and myeloid cells (Figure 10D; Supplementary Figure S3C). Among them, both ERCC6L and MYB had significant differences in T cells (Figure 10E; Supplementary Figure S3D).

**FIGURE 11:**
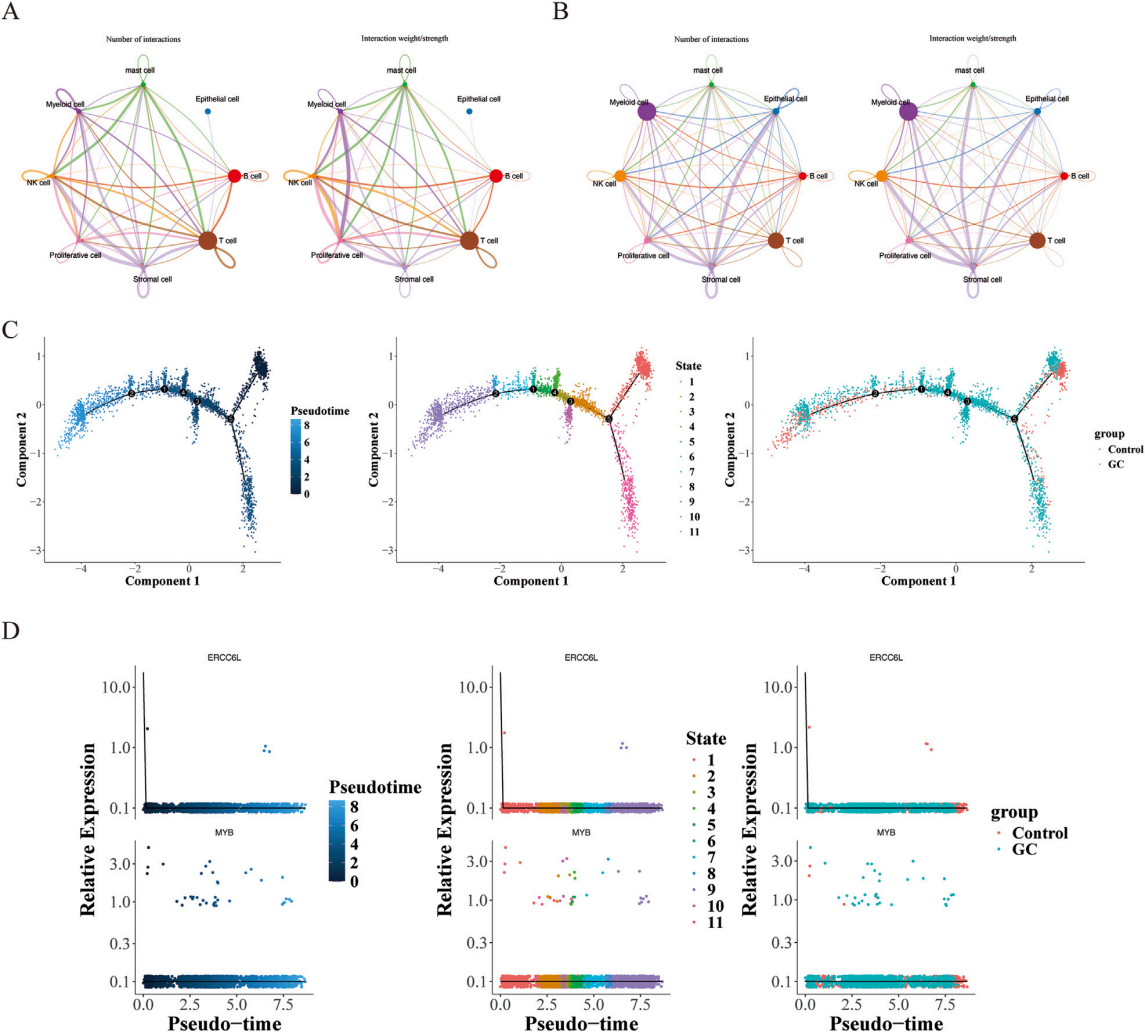
Cell communication and pseudotime-sequence analysis. **(A)** Cell communication analysis in the normal group. The left side was plotted by number, and the right side was plotted by weight. Different colors represented different cell types. The thickness of the lines represented the number/intensity of cell-cell interactions. The thicker the line, the more/stronger the cell-cell interactions. **(B)** Cell communication analysis in the tumor group. **(C)** Cell differentiation trajectory graph. Each dot in the graph represented a cell. The numbers in the black circles represented the nodes for determining different cell states in the trajectory analysis, and the color shade was used to represent the order of pseudotime. **(D)** Dynamic expression profiles of key genes in T-cells.

Cell-cell communication mediated by ligand-receptor complexes is crucial for coordinating various biological processes such as development, differentiation, and inflammation. Cell communication analysis showed that in the control group, the Proliferative cell-NK cell interaction was dominant, while in the GC group, the Stromal cell-NK cell interaction was the strongest. It is worth noting that in both groups, the number of T cells was relatively large (Figures 11A,B). In the training set, ERCC6L and MYB were significantly positively correlated with T cells CD4 memory activated; and in the single-cell data, both ERCC6L and MYB had significant differences in T cells, so we selected T cells as the key cells. Clustering analysis of the top 20 principal components in T cells revealed 12 subgroups (Supplementary Figure S4), and their cell differentiation trajectories are shown in Figure 11C. With the development of time, the expression of the MYB gene first decreased, then increased and then decreased again, and fewer cells expressed the ERCC6L gene in T cells (Figure 11D).

### 3.11 Verification of gene expression from cells and tissues

**FIGURE 12:**
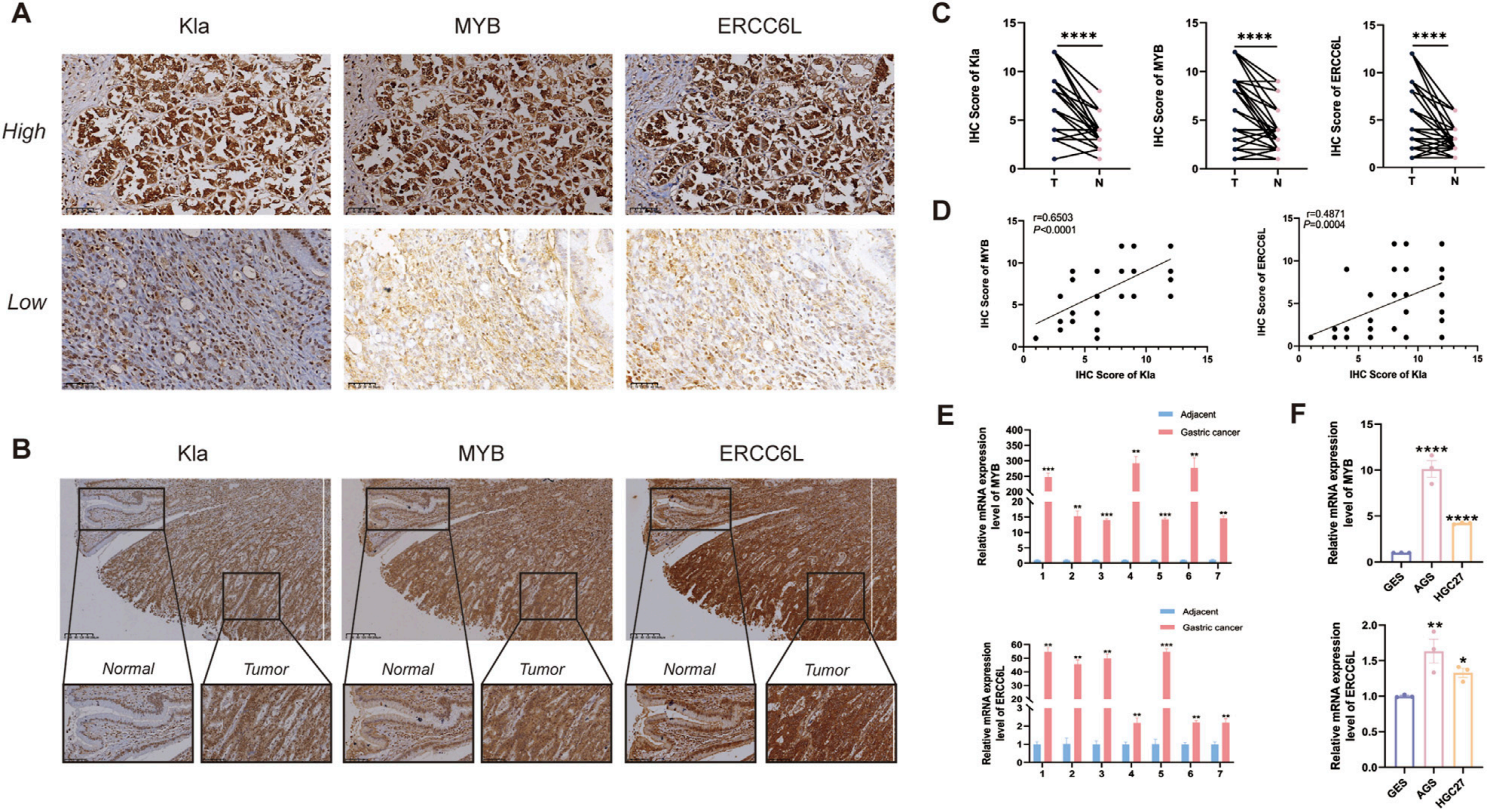
Validation of gene expression from cells and tissues. **(A)** Immunohistochemical analysis of Kla, MYB, and ERCC6L protein expression in gastric carcinoma samples. **(B, C)** Comparative immunohistochemistry of gastric cancer tissues and matched adjacent non-tumorous tissues, including representative images and statistical summaries (n = 48). “****” represented *p* < 0.0001. **(D)** Linear regression and Pearson correlation analyses evaluating the relationship between Kla and MYB/ERCC6L expression in tumor tissues. **(E)** qPCR detection of ERCC6L and MYB expression in gastric cancer samples (n = 7), the results showed that the expression in gastric cancer tissues (T) was higher than that in normal tissues (N). ***p < 0.001. **(F)** RT-qPCR results confirming MYB and ERCC6L transcript levels in AGS, HGC-27 and GES-1 cell lines. “**” represented *p* < 0.01, and “****” represented *p* < 0.0001.

Immunohistochemical analysis showed intensified staining of ERCC6L, MYB, and Kla in GC tissues relative to adjacent normal tissues, as depicted in representative images (scale bar: 200 μm) (Figures 12A,B). Quantitative evaluation using staining scores (range: 0–12) confirmed significantly higher protein expression levels in GC specimens compared to matched non-tumor controls (n = 48, *p* < 0.001) (Figure 12C). Correlation analysis revealed a positive association between ERCC6L and Kla, as well as between MYB and Kla (Figure 12D). We conducted a qPCR experiment on 7 pairs of cancerous and adjacent tissues, finding that ERCC6L and MYB expression was significantly higher in gastric cancer tissues (Figure 12E). *In vitro* experiments demonstrated elevated mRNA expression of ERCC6L and MYB in human gastric adenocarcinoma AGS and HGC-27 cells compared to normal gastric epithelial GES-1 cells (Figure 12F).

## 4 Discussion

GC remains a significant global health burden, driven by a multifactorial etiology involving dietary patterns, *H. pylori* infection, and environmental exposures (Smyth et al., 2020). Emerging evidence suggests that tumor-derived metabolites modulate peripheral immune cell function, thereby impairing antitumor immunity. In particular, lactic acid metabolism has garnered attention for its role in tumor bioenergetics and immune evasion (Yang et al., 2022). Protein lactylation, a modification mediated by lactic acid, facilitates the polarization of macrophages toward the immunosuppressive M2 phenotype, thereby dampening antitumor immune responses within the TME (Fan et al., 2023; Piao et al., 2022). Although individual studies have explored macrophage polarization and protein lactylation in cancer, no prior research has integrated these pathways using bioinformatic approaches to identify prognostic genes in GC. Addressing this gap may provide a theoretical framework for future clinical applications.

This study leveraged GC transcriptomic data from TCGA to identify 428 genes linked to both protein lactylation and macrophage polarization, distinguishing expression profiles between tumor and normal tissues. Through univariate Cox regression and machine learning algorithms, MYB and ERCC6L were identified as prognostically relevant genes. A predictive model was subsequently constructed to evaluate their clinical utility, offering novel insights into risk stratification and therapeutic response prediction in GC.

This study using the ssGSEA algorithm found that gastric cancer tissues exhibit a high PLRGs score and low MPRGs score, highlighting a synergy between metabolism and immune regulation. The elevated GSVA score for lactylation-related genes suggests these genes are upregulated in gastric cancer, indicating lactylation’s potential role in the disease’s progression (Sun et al., 2024). Additionally, the low macrophage score in gastric cancer implies a decrease in anti-tumor M1 macrophages and an increase in tumor-promoting M2 macrophages (Ge and Wu, 2023). Elevated lactate levels indicate increased glycolytic metabolism in tumor cells, promoting gastric cancer progression through two mechanisms. First, lactate acts as a metabolic signal, activating cancer-promoting genes like MYC via histone lactylation modification and enhancing DNA repair gene expression, leading to chemoradiotherapy resistance (Zhang et al., 2019; Sun et al., 2025). Second, lactate induces macrophages to polarize to the M2 phenotype via the ERK/STAT3 pathway (Mu et al., 2018). M2 macrophages express factors like arginase-1 and VEGFA, promoting angiogenesis, immunosuppression, and metastasis, while reducing the proportion of anti-tumor M1 macrophages (Ge and Wu, 2023). The abnormal activation of the “metabolism-appearance-immunity” axis creates a vicious cycle in the gastric cancer microenvironment, where high lactylation boosts M2 polarization, which in turn sustains metabolic abnormalities. The ERCC6L and MYB genes could serve as prognostic markers by strengthening the link between lactylation modification and M2 polarization, offering a new target for anti-tumor strategies aimed at the “lactate metabolism-macrophage polarization” axis.

In this study, MYB and ERCC6L were identified as prognostic genes. ERCC6L, which encodes an ATP-dependent DNA helicase, is involved in DNA tension sensing and Holliday junction branch migration (Baumann et al., 2007; Biebricher et al., 2013). It has cancer promoting activity in a variety of cancers, such as promoting the progression of hepatocellular carcinoma by activating PI3K/Akt and NF-κB signaling pathways (Chen et al., 2020). It is noteworthy that the activation of PI3K/Akt pathway can reshape the glucose metabolism state of cells (Wang et al., 2024), and its end product lactic acid can mediate histone lactation modification, and then regulate gene expression (Liberti and Locasale, 2020), which suggests that ERCC6L may indirectly affect histone lactation level through PI3K/Akt metabolic reprogramming axis, and then reshape the tumor microenvironment. In addition, the NF-κB pathway activated by ERCC6L may promote the polarization of pro-inflammatory M1 macrophages (Li et al., 2023). On the other hand, it may indirectly induce M1/M2 polarization of macrophages by affecting the metabolism and cytokine secretion of tumor cells (Shen et al., 2023). However, it is worth noting that the univariate Cox regression analysis of this study showed that its high expression was associated with the longer survival time of patients (HR = 0.664), suggesting that it has both cancer promoting and tumor inhibiting functions, similar to the “double-sided” genes such as SPDEF (Ye et al., 2020; Shen et al., 2018). At the same time, the expression analysis results of ERCC6L in the training set and validation set were consistent with the results of RT-qPCR, and the expression level was high in tumor cells, which further verified the reliability of the results, and its dual role also suggested that ERCC6L might become a potential target. In conclusion, the functional contradiction of ERCC6L gene in tumorigenesis and development needs to be further explored in order to develop treatment strategies.

MYB is a transcription regulator crucial for hematopoietic regulation and is classified as an oncogene due to its link to leukemia and lymphoma. It regulates the proliferation and differentiation of hematopoietic progenitor cells. The CD36-BATF2/MYB trait predicts anti-PD-1 response in gastric cancer (GC) (Jiang et al., 2023; Ducka et al., 2021). This study found high MYB expression in GC tissues, with poor prognosis linked to low expression, similar to breast cancer. However, MYB’s specific role in GC, particularly regarding cell proliferation, metastasis, and immune escape, remains unclear and requires further research (Knopfova et al., 2018). Overall, ERCC6L and MYB have significant biological and clinical roles in various tumors, but their expression and prognostic value differ by tumor type. Studying their mechanisms in various tumors and interactions with genes and pathways is crucial for advancing tumor diagnosis and treatment strategies.

The dual gene risk model based on ERCC6L and MYB showed significant prognostic stratification ability in the training set (TCGA-GC) and validation set (GSE66229). This model only needs to calculate the risk score through the expression level of two genes. Compared with polygene panel or whole transcriptome analysis, it may have higher economic benefits and certain clinical transformation potential. The high expression of ERCC6L and MYB may be associated with the improvement of patients’ survival, suggesting that ERCC6L and MYB may play a potential protective role by inhibiting tumor progression or enhancing treatment sensitivity. This hypothetical biological mechanism has also been preliminarily verified in the experiment: the expression levels of the two genes may be related to lactate metabolism in the tumor microenvironment (verified by Kla modification) and M2 macrophage polarization (immune infiltration analysis), revealing the mechanism of immune metabolism regulation behind the model. Although the model contains only two genes, its predictive efficiency (AUC>0.6) is equivalent to some more complex molecular models (Sun et al., 2024), and it can predict the recurrence risk independently of TNM staging. This may provide a potential tool with simplicity, operability and mechanism explanation for the individualized diagnosis and treatment of gastric cancer.

The cancer recurrence rate was notably higher in the high-risk group than in the low-risk group (*p* < 0.05), highlighting the model’s ability to predict survival outcomes and reflect tumor behavior differences. Although the model’s AUC value wasn't very high, its stability in validation and analysis with clinical features like T stage and age suggests potential clinical use. The nomogram-based prognosis system combines risk scores with clinical indicators for better patient management in gastric cancer. Future large-scale studies and experiments are needed to confirm the model’s applicability.

The TME, widely recognized for its critical role in cancer progression—including proliferation, invasion, and metastasis (Arner and Rathmell, 2023)—showed a strong association with the identified prognostic signature. Elevated immune cell infiltration was observed in the high-risk group, particularly involving memory B cells, resting memory CD4^+^ T cells, Tregs, monocytes, resting dendritic cells, and resting mast cells. In GC, immune cell dynamics substantially influence tumor progression. The differentiation of activated memory CD4^+^ T cells has been linked to patient prognosis (Sun et al., 2023), while memory B cells contribute to the formation of tertiary lymphoid structures (TLS), which correlate with favorable immunotherapy responses in solid tumors (Hu et al., 2024). Tregs undermine antitumor immunity and facilitate tumor progression in GC (Negura et al., 2023). Monocytes recruited into the TME frequently differentiate into M2 macrophages, promoting tumor growth (Xu et al., 2022). Although dendritic cells are expected to prime T-cell responses, their immunostimulatory functions are often suppressed within the TME, diminishing immune surveillance (Heras-Murillo et al., 2024). Mast cells, associated with increased microvessel density and tumor-associated macrophage markers, are inversely correlated with patient survival (Eissmann et al., 2019). Collectively, these immune components contribute to immune evasion and metastatic progression.

Chemoresistance remains a major obstacle in the treatment of GC, significantly contributing to its elevated mortality rate (Zen et al., 2022). Stratifying patients based on chemosensitivity may optimize therapeutic efficacy by identifying subpopulations more likely to benefit from standard chemotherapy. In the present study, individuals classified as high-risk demonstrated increased sensitivity to AZD.0530, CCT007093, DMOG, JNJ-26854,165, and LFM-A13. AZD.0530, originally developed as a tyrosine kinase inhibitor, has undergone multiple clinical trials with limited success across various malignancies (Li et al., 2024b; Hennequin et al., 2006). CCT007093 acts as a selective WIP1 phosphatase inhibitor (Buss et al., 2012). Dimethyloxalylglycine (DMOG) functions by nonspecifically stabilizing hypoxia-inducible factor 1, as shown in numerous preclinical models (Imran Khan, 2022). JNJ-26854,165 is a novel chemotherapeutic agent that activates p53 and concurrently inhibits human double minute protein 2 (HDM2) (Chargari et al., 2011). LFM-A13, the first small-molecule inhibitor of Bruton’s tyrosine kinase (BTK), has demonstrated efficacy in curbing tumor growth (Mahajan et al., 1999), with additional anti-proliferative and pro-apoptotic effects observed in breast cancer models (Rozkiewicz et al., 2020). Currently, these drugs lack enough clinical validation for GC treatment. More studies are needed to confirm their effectiveness and safety, aiming to offer precise, personalized treatment for high-risk patients.

In conclusion, this study introduces a novel prognostic signature based on protein lactylation and macrophage polarization, capable of reliably predicting outcomes in GC. The findings establish a mechanistic and predictive foundation, highlighting the relevance of protein lactylation and macrophage polarization in the prognostic landscape of GC. This work may inform future development of targeted clinical interventions. Our study has several limitations: a single Kla gene doesn't fully represent the lactylation modification network; secondly, although the potential roles of ERCC6L and MYB in the association between emulsification modification and macrophage status were found, the mechanism hypothesis in the current study was still speculative and lacked direct functional evidence; and despite an AUC value over 0.65, the model’s accuracy and discrimination need improvement. In the future, we plan to use three cell lines to construct *in vitro* validation, overexpression/gene knockout and other functional experiments to verify the existing hypothetical mechanisms, and further analyze the specific molecular mechanisms of ERCC6L and MYB genes in emulsification modification and macrophage state regulation.

## Data Availability

The original contributions presented in the study are included in the article/Supplementary Material, further inquiries can be directed to the corresponding authors.
