# Supplementary material for: Prognostic and tumor microenvironmental features of gastric cancer revealed by macrophage polarization and protein lactylation-related genes
Source: Front Genet. 2025 Jul 2;16:1541489. doi: 10.3389/fgene.2025.1541489 (PMC12263385; doi:10.3389/fgene.2025.1541489)
Supplement: Supplementary file 3 [file DataSheet1.pdf]

Supplementary Material

1 Supplementary Figures

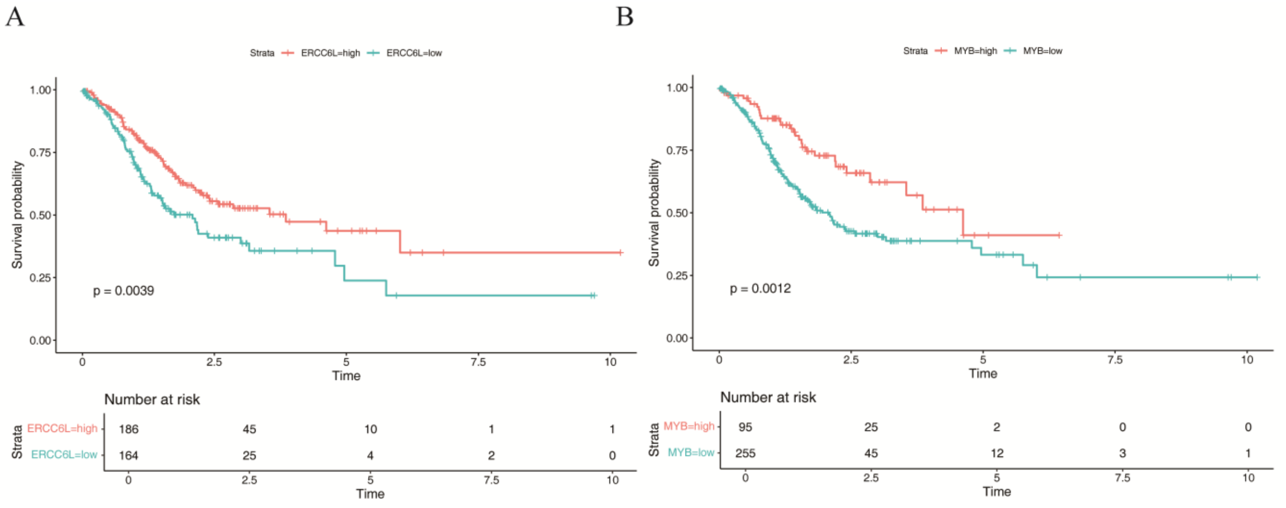

Supplementary Figure 1. Kaplan-Meier survival curve analysis of key genes. (A) Kaplan-Meier survival curve of the ERCC6L gene. (B) Kaplan-Meier survival curve of the MYB gene.

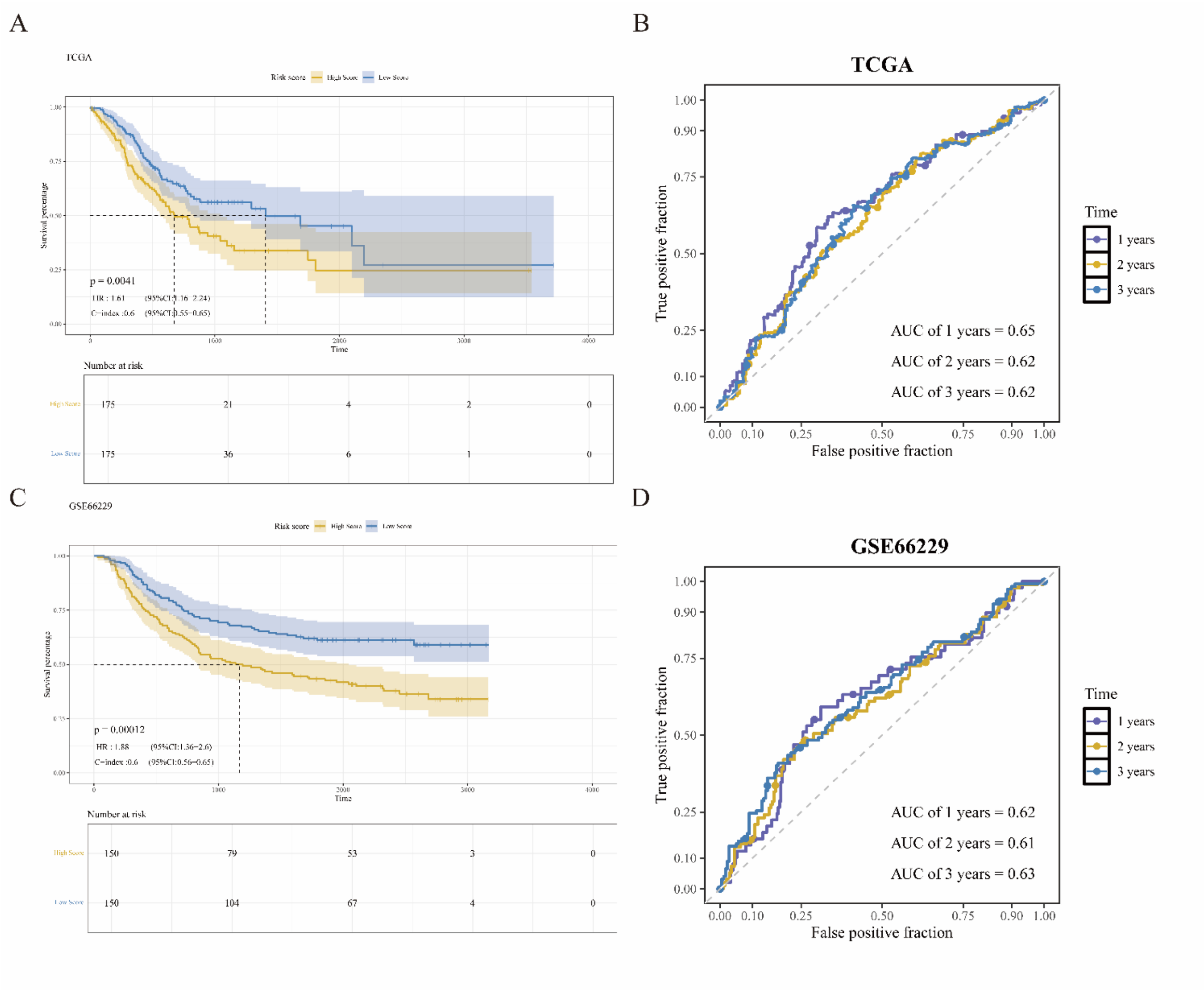

Supplementary Figure 2. KM and ROC curves for Sun et al.'s gene models. (A) KM curve for the COL4A1, SLC16A7, and IRAK1 model in the TCGA-GC dataset. (B) ROC curve for the COL4A1, SLC16A7, and IRAK1 model in the TCGA-GC dataset.(C)KM curve for the COL4A1, SLC16A7, and IRAK1 model in the GSE66229 dataset. (D)ROC curve for the COL4A1, SLC16A7, and IRAK1 model in the GSE66229 dataset.

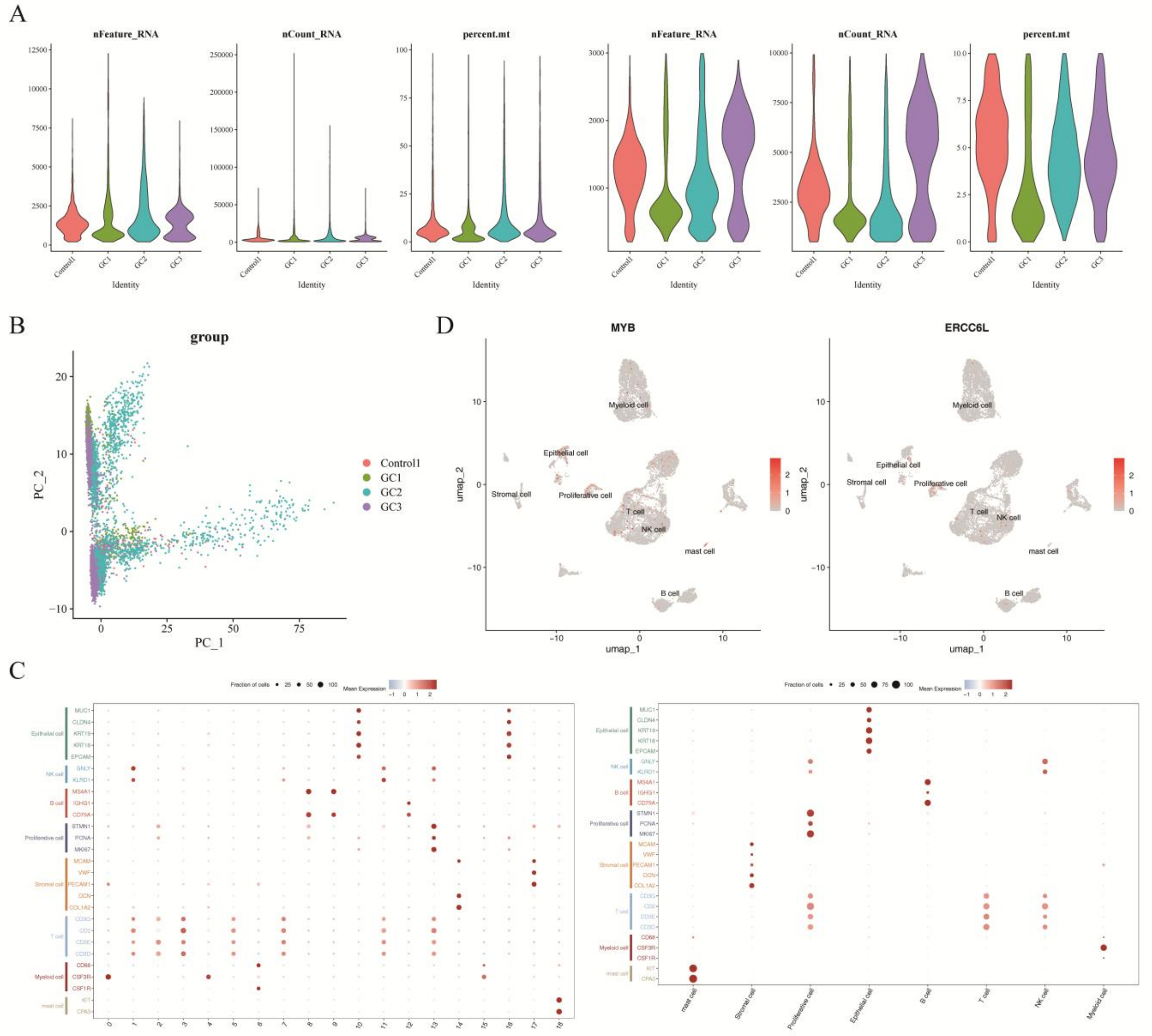

Supplementary Figure 2. Single-cell analysis to identify key cells. (A) Violin plots of nFeature_RNA, nCount_RNA, and percent_mt before and after quality control. nFeature_RNA represents the number of gene expressions in a cell; nCount_RNA represents the count of genes; percent_mt indicates the proportion of mitochondria, and the size of the mitochondrial proportion reflects the degree of cell damage. (B) PCA sample cell distribution map. Each dot represents a cell, and different colors represent different samples. (C) Cell marker dot plot. Upper figure: The expression of markers in each cluster. Lower figure: The expression of markers in each cell cluster. (D) The expression of core genes in different cells.

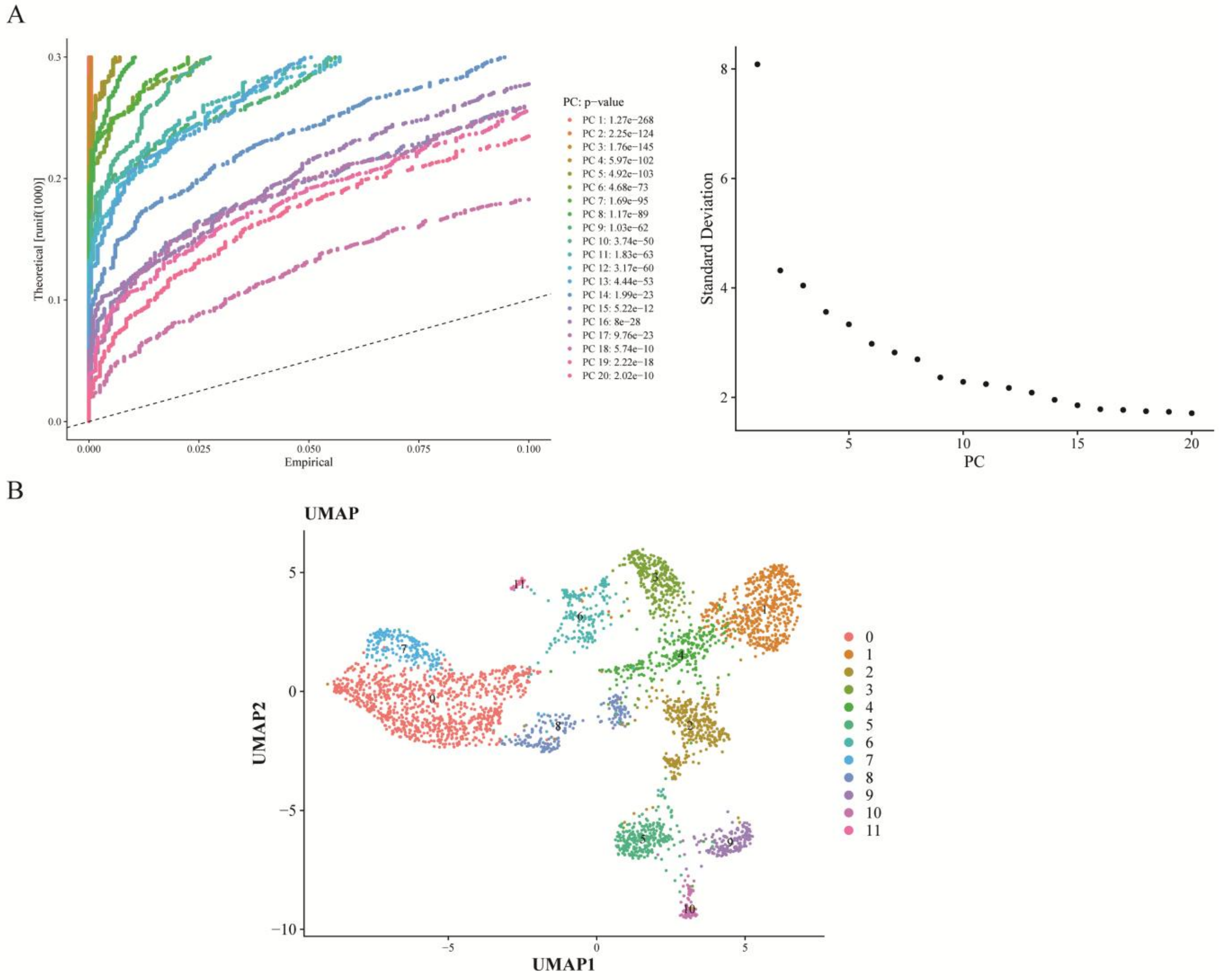

Supplementary Figure 3. Result graphs of T-cell dimensionality reduction and clustering. (A) Principal component analysis of T-cells. JackStraw graph (left) and principal component inflection point graph (right). (B) UMAP graph of T-cell sub-population distribution.
